# Breast Cancer Stem Cells Upregulate IRF6 in Stromal Fibroblasts to Induce Stromagenesis

**DOI:** 10.3390/cells13171466

**Published:** 2024-08-31

**Authors:** Harshini Muralidharan, Thomas Hansen, Anja Steinle, David Schumacher, Elmar Stickeler, Jochen Maurer

**Affiliations:** 1 Department of Obstetrics and Gynecology, University Hospital Aachen (UKA), 52074 Aachen, Germany; 2 Department of Anesthesiology, University Hospital, RWTH Aachen University, 52074 Aachen, Germany; 3 Department of Nephrology and Clinical Immunology, RWTH Aachen University, 52074 Aachen, Germany; 4 Center for Integrated Oncology (CIO), Aachen, Bonn, Cologne, Düsseldorf (ABCD), Venusberg-Campus 1, 53127 Bonn, Germany

**Keywords:** IRF6, triple-negative BCSC, BCSC niche, BCSC–fibroblast interaction, CAF, fibrosis, tumor stroma, stromagenesis

## Abstract

The microenvironment of a cancer stem cell (CSC) niche is often found in coexistence with cancer-associated fibroblasts (CAFs). Here, we show the first in-depth analysis of the interaction between primary triple-negative breast cancer stem cells (BCSCs) with fibroblasts. Using 2D co-culture models with specific seeding ratios, we identified stromal fibroblast aggregation at the BCSC cluster periphery, and, on closer observation, the aggregated fibroblasts was found to encircle BCSC clusters in nematic organization. In addition, collagen type I and fibronectin accumulation were also found at the BCSC–stromal periphery. MACE-Seq analysis of BCSC-encapsulating fibroblasts displayed the transformation of stromal fibroblasts to CAFs and the upregulation of fibrosis regulating genes of which the Interferon Regulatory Factor 6 (*IRF6*) gene was identified. Loss of function experiments with the *IRF6* gene decreased fibroblast encapsulation around BCSC clusters in 2D co-cultures. In BCSC xenografts, fibroblast IRF6 expression led to an increase in the stromal area and fibroblast density in tumors, in addition to a reduction in necrotic growth. Based on our findings, we propose that fibroblast IRF6 function is an important factor in the development of the stromal microenvironment and in sustaining the BCSC tumor niche.

## 1. Introduction

Breast cancer is a highly heterogenous disease and is the most commonly diagnosed malignancy worldwide [1,2]. Among the molecular subcategories that have been used to classify breast tumor heterogeneity, triple-negative breast cancer (TNBC) has been characterized as the most aggressive subtype [3,4]. TNBCs manifest as grade 3 carcinomas with poor differentiation, central necrosis, desmoplastic stroma, abnormal extracellular matrix (ECM) remodelling in the tumor microenvironment (TME), a high recurrence rate, and poor survival [5,6,7,8,9]. The tumorigenicity of TNBC is, in part, exercised by BCSCs that possess the characteristic properties of self-renewal, cellular plasticity, and tumor initiation; furthermore, they can induce metastasis, recurrence, and therapy resistance [10,11,12,13,14,15,16].

CSCs reside in evolved niches of the TME [17,18]. CAFs and their reciprocal ECM deposition has emerged as one of the main stromal elements in the CSC niche [19,20,21]. In invasive mammary carcinomas, cancer cells recruit and transform fibroblasts to CAFs, which are observed to be the most dominant cellular component in the TME [22,23]. Data from tumor imaging reveal CAFs to be spatially oriented in close proximity to tumor cell nests where they circumferentially align in a longitudinal pattern at the border of cancer cell clusters [24,25]. CAFs have also been implicated in inducing the desmoplastic response or tumor fibrosis in the breast cancer microenvironment where they are reported to synthesize and deposit abnormal amounts of ECM proteins, mainly collagen type I and fibronectin [26,27,28]. In addition, CAFs have been shown to influence stemness, proliferation, and, potentially, metastasis in breast cancer [8,20,29,30,31]. Literature on the BCSC-CAF interaction is still sparse; therefore, research was undertaken to model and characterize the BCSC niche in the presence of stromal fibroblasts.

To model the BCSC/fibroblast TME, previously profiled primary triple-negative BCSCs [32,33,34] were co-cultured with fibroblasts. Being the principal element in the breast TME, the fibroblast seeding number was made higher than BCSCs. The analysis of the 2D co-culture models with increased fibroblast ratios revealed the encapsulation of the BCSC clusters by dense fibroblast aggregates at the tumor–stromal interface. Furthermore, fibroblast encapsulation resulted in the enhanced accumulation of ECM proteins: collagen type 1 and fibronectin at the BCSC cluster periphery. Investigation of the differentially expressed genes (DEGs) in aggregated fibroblasts surrounding BCSC clusters observed enrichment in CAF characteristics and fibrosis regulation.

IRF6 transcription factor, a significantly upregulated DEG, was among the fibrosis-regulating genes that was influenced by BCSCs. In fibroblasts, IRF6 has been previously associated with the regulation of morphology, differentiation, cell adhesion, and ECM deposition in the context of wound-healing and van der Woude syndrome [35,36]. In the BCSC/fibroblast 2D co-cultures, IRF6 reduction resulted in decreased fibroblast encapsulation around BCSC clusters. In BCSC xenograft tumors grown with IRF6 knockdown fibroblasts, a reduction in stromal area and fibroblast density was documented, with an increase in necrotic growth. 

## 2. Materials and Methods

### 2.1. Cell Culture

TNBC stem cell lines were previously isolated from patient tumor samples post chemotherapy [32,33,34]. BCSC lines were expanded in 2D culture dishes (6 cm) and was split once a week. Confluent dishes were washed with 1× DPBS (Gibco, 14200-067, Paisley, UK) and incubated with Accutase (Sigma-Aldrich, A6964, St. Louis, MO, USA) for 20 min at 37 °C. The cell mixture was diluted with MEBM (Lonza, CC-3151, Basel, Switzerland) in a ratio of 1:1 and detached cells were pelleted by centrifugation at 200× *g* for 3 min. Cell pellet was resuspended in 1 mL mammary stem cell (MSC) medium [32], and counted using Neubauer chamber. Seeding density of 1.2 × 10^5^ cells were added to 1 mL ice-cold MSC medium containing 2% Matrigel and spread evenly onto a new dish. Dishes were incubated at 37 °C for 20 min for solidification of the Matrigel, after which 4 mL of MSC medium was added. The dishes were maintained in a hypoxic chamber with a gas composition of 92% N_2_, 5% CO_2_, and 3% O_2_. Culture dishes were supplemented with fresh medium twice a week. BCSC cultures with a passage number of 35 and above were not used for experiments. BCSC lines were authenticated every six months using multiplex human cell authentication test. 

Human dermal fibroblasts (MTI GlobalStem, GSC-3002) were expanded in a T75 flask with 15 mL DMEM (Gibco, 41966-029, Grand Island, NY, USA) containing 10% FBS (Gibco, 10500-064) and 1% Penicillin–Streptomycin (Gibco, 15140-122). Fibroblast culture dishes were maintained in normoxia at 37 °C. Medium was changed twice a week. The dish was split when culture was 70% confluent. Cells were split using Accutase, washed, and 2.5 × 10^5^ cells/dish were reseeded. Fibroblast cultures with a passage number of 8 and above were not used for experiments.

### 2.2. BCSC/Fibroblast Co-Culture

BCSC and fibroblast monocultures were harvested and pelleted. Cell pellets were resuspended individually in MSC medium and were counted. Based on the cell-culture dish and the total cell seeding number of the dish (96-well plate [Total cell seeding number: 3600 cells] or 100 mm dish [Total cell seeding number: 500K–600K cells]), BCSCs and fibroblasts were mixed in a ratio of 1:10 or 1:2 in MSC medium with 2% Matrigel in Eppendorf tubes. Total seeding volume used for 96-well plates and for 100 mm dishes is 20 µL/well and 1 mL/dish, respectively. Cell mixture for a 100 mm dish was pipetted in the center of the dish and spread evenly along the surface using a pipette tip. Care was taken to avoid spreading the cell mixture too close to the edge to prevent accumulation of cells at the corners. Dishes were incubated at 37 °C for 30 min for solidification of the Matrigel after which MSC medium was added (120 µL/well and 8 mL–10 mL/dish). The dishes were maintained in a hypoxic chamber for a period of 10 days. Medium was replaced with fresh MSC medium once every three days until the culture has grown for 7 days, after which medium was changed every day. 

### 2.3. Lentiviral Production and Transduction

Lenti-PGK-pLV-mCherry (Addgene, 36084), Lenti-PGK-H2B-EGFP (Addgene, 21210), Control Scrambled Vector: pLV[shRNA]-mCherry/Neo-U6>Scramble_shRNA#1 (Vectorbuilder, VB010000-0012tsh), IRF6 Knockdown Vector: pLV[shRNA]-mCherry/Neo U6>hIRF6[shRNA#1] (Vectorbuilder, VB900120-6695mnh), and IRF6 Overexpression Vector: pLV[Exp]-mCherry/Neo-EF1A>hIRF6[NM_006147.4] (Vectorbuilder, VB900123-7592acs) were obtained in the form of agar stabs. Bacteria was amplified and plasmids were extracted using the Macherey Nagel NucleoBond^®^ Xtra maxi plus kit (12748412, Leicestershire, UK). 

Lentiviruses were produced using HEK293T (Invitrogen, RRID: CVCL_6911, Thermo Fisher, Waltham, MA, USA) cells in 6-well plates (Falcon, Corning, 353046, Durham, NC, USA). In an Eppendorf tube, the helper packaging mix was prepared using pCMVdR 8.74 (Addgene, 22036, Watertown, MA, USA; 700 ng/well) and pMDVSVG (Addgene, 8454, Watertown, MA, USA; 350 ng/well). In a different tube, 4 µL/well of X-tremeGENE^TM^ HP DNA Transfection Reagent (Sigma-Aldrich, 6366236001, Taufkirchen, Germany) was combined with 400 µL/per well of Opti-MEM (Life Technologies, 11058021, Carlsbad, CA, USA), gently mixed, and added to the helper plasmid mix. Lenti construct plasmid was spotted in the middle of each well with a concentration of 1000 ng/spot. Then, 400 µL/well of helper plasmid mixture was added to the spot and the plate was incubated for 30 min at room temperature. 2 × 10^6^ HEK293T cells suspended in 1600 µL of DMEM + FBS + P/S media (described above) was added to the well. After 24 h, the culture medium was replaced with 2 mL/well of UltraCULTURE^TM^ (Lonza, 12-725F, Basel, Switzerland). An additional 24 h later, the viral soup was collected from all wells and centrifuged at 500 x g for 10 min at room temperature. The supernatant was filtered through a 0.45 µm filter to remove suspended HEK293T cells. 

For fluorescence tagging, 50% confluent BCSC and fibroblast cultures grown in 6-well plates were emptied of media and 330 µL of filtered viral soup was added to each well together with a 100 µL/well of media substituted with 15 µg/mL concentration of polybrene (Sigma-Aldrich, 107689, Taufkirchen, Germany). The plates were centrifuged at 1000× *g* for 1 h at room temperature and then incubated in hypoxia (BCSC) or normoxia (fibroblast) for 24 h. Finally, the cell culture plates were washed 3 times with 1× DPBS and fresh cell-specific medium (MSC or DMEM + FBS + P/S) was added. Fresh UltraCULTURE^TM^ medium was added to HEK293T cells for production of Day 2 of viral soup. BCSC lines were made to undergo a second round of transduction by following the above steps. Transduced BCSC lines were expanded and sorted using FACS. For fibroblasts, the cells were allowed to expand, followed by an antibiotic selection of transduced cells. Selected cells were monitored in terms of proliferation and growth. 

IRF6 control, knockdown, and overexpression lentivirus produced were pelleted by adding a 10% sucrose-containing buffer [37] over the viral soup at a 4:1 ratio followed by centrifugation of the mixture at 10,000× *g* at 4 °C for 4 h. Pellets were resuspended in 1 mL of UltraCULTURE^TM^ medium. The virus was titrated and an MOI of 5 was used to transduce fibroblasts. Antibiotic selection was performed to sort transduced cells. 

### 2.4. Flow Cytometry

To analyze BCSC-influenced fibroblasts, BCSCs and fibroblasts were seeded in a 1:2 ratio in a 100 mm dish and cultured for 10 days. Cells were detached, suspended in MSC medium, and passed through a 40 µm filter into a FACS tube. FACS sort was performed based on the fluorescent marker of BCSCs (EGFP) and fibroblast (RFP) using an Aria II Cytometer with 3 lasers (violet, blue, and red) and analyzed with BD FACSDiva software 8.0.1. 

### 2.5. Rapid MACE-Seq

Three CF population samples from each BCSC line were analyzed. Cell pellets of sorted fibroblasts or harvested control fibroblasts were gently resuspended in 200 µL of ice-cold RNA*later^®^* (Sigma-Aldrich, R0901, Waltham, MA, USA). The following sequence of steps was performed by the team in IZKF, Uniklinik RWTH Aachen. RNA isolation was performed using simply RNA Tissue Kit and miRNA Tissue Kit according to the manufacturer’s instructions with the Maxwell RSC instrument from Promega. Total extracted RNA was analyzed with the Quantus Fluorometer (Promega, Madison, WI, USA). RNA quality control was carried out with the Agilent Tapestation 4200 system (Agilent Technologies, Inc., Santa Clara, CA, USA). An RNA Integrity Number (RIN) of at least 8.9 verified the high quality of all RNA samples. Sequencing libraries were generated from 100 ng of total RNA using the Rapid MACE-seq library preparation kit as described by the manufacturer (GenXPro, Frankfurt Main, Germany). The libraries were run on an Illumina NextSeq500 platform (Illumina, San Diego, CA, USA) using Single-Read sequencing on a High Output 75 cycles Kit (Illumina, San Diego, CA, USA). FASTQ files were generated using bcl2fastq (Illumina, San Diego, CA, USA). Quality control was performed using FastQC (v0.6.7) [38]. No significant irregularities were found. The reads were aligned to the GRCh38 reference genome utilizing STAR (v2.5.2b) [39]. The quantification of genes was performed with FeatureCounts from the SubRead package (v1.5.1) [40]. Multimapping reads were counted as fractions. Differential expression analysis was conducted using DESeq2 [41] with default parameters. PCA analysis was conducted using the function plotPCA from the DESeq2 package (v.1.40.2) after running standard variance stabilization transformation procedure.

Pathway analysis of BCSC1 CF and BCSC2 CF datasets was performed using GSEA_4.1.0. Gene Ontology (GO) enrichment analysis of the 55 genes was performed using GOnet [42]. Fibrosis-related genes were identified from the CTD Gene–Disease Associations dataset in Harmonizome 3.0 [43]. 

### 2.6. RNA Preparation and qPCR

BCSC + Fi co-cultures grown in 1:2 ratio was sorted for fibroblasts using FACS. Extraction and purification of total RNA was performed using the RNeasy Mini Kit (Qiagen, 1038703, Hilden, Germany) according to the manufacturer’s instructions. Isolated RNA was quantified using NanoDrop One. Reverse-transcription of isolated RNA to complementary DNA was performed using the EvoScript Reverse-Transcriptase Kit (Roche, 07912323001, Mannheim, Germany). RT-PCR was performed using the SYBR Green Master Mix (Bio-Rad Laboratories, 1725150, Hercules, CA, USA) and IRF6 mRNA was detected using LightCycler 480 (Roche Life Science). IRF6 primer: 5′-CAAAACTGAACCCCTGGAGATGGA-3′; 3′-CCACGGTACTGAAACTTGATGTCC-5′. Beta-actin primer (control): 5′-CCAACCGCGAGAAGATGA-3′; 3′-CCAGAGGCGTACAGGGATAG-5′.

### 2.7. Immunofluorescence Staining

Cultures were grown in 96-well plates (Greiner Bio-One, 655090, Kremsmünster, Austria). Cells were fixed with ice-cold methanol for 15 min. Fixed cells were washed with DPBS and permeabilized with 1× TBST for 6 min. The plate was washed with fresh 1 mg/mL ovalbumin in DPBS (ova/DPBS) and blocked with 50 µL/well ova/DPBS for 1 h at 37 °C. Primary antibody (anti-COL1A1: Atlas Antibodies, Stockholm, Sweden, HPA011795; anti-FN1: abcam, ab2413, Cambridge, MA, USA; anti-IRF6: Atlas Antibodies, HPA063121, anti-RFP: SICGEN, AB1140-100, Catanhede, Portugal) was diluted in ova/DPBS to the required concentration and 50 µL/well was added. The plate was covered with moistened tissue before placing the lid and was incubated overnight at 4 °C. After 12 h, cells were washed with DPBS and incubated with 50 µL/well of secondary antibody (Alexa Fluor 568: Thermo Fisher Scientific, A-11057; Alexa Fluor 647: Thermo Fisher Scientific, A-31573, Waltham, MA, USA) for 1 h at 37 °C. The plate was washed with DPBS and incubated for 10 min with DAPI constituted nuclei staining buffer. To avoid DAPI-induced background fluorescence, the solution was removed and cells were washed with DPBS. Finally, 200 µL/well nuclei staining buffer was added and the plate was stored at 4 °C. 

Confocal images were captured using Zeiss LSM 710 (Carl Zeiss GmbH, Munich, Germany) and analyzed using ZEN blue 3.2 and Image J 1.53v. Cells in co-culture were distinguished on the basis of their fluorescent label: BCSC (EGFP) and fibroblast (RFP). Phenotype counts were measured using confocal images and analyzed for fibroblast aggregation at the BCSC cluster periphery in at least 3 regions. Phenotypic frequency was derived as a percentage by comparing the phenotype count to the total number of BCSC clusters in co-culture. 

COL1A1 and FN intensities were measured using mean gray-value in defined areas (established using macros, Image J) in the CF section of the co-cultures. COL1A1 intensity was measured across 40 regions and FN intensity was measured across 70 regions at the CF and NCF populations of the co-culture. IRF6 expression in the BCSC CF section of the co-culture was measured by dividing the integrated density of staining in an image by the total number of nuclei in that image. 

### 2.8. Immunohistochemistry

In vitro H&E staining: The procedure was improvised by Lab Technician Anja Steinle of the Molecular Gynecology Laboratory, Uniklinik RWTH Aachen. Co-cultures grown in 35 mm dishes were washed with 1× DPBS and fixed with ice-cold methanol for 20 min. Then, 1 mL of 100% ethanol was added to the dishes, gently swirled and incubated for 3 min. Ethanol wash was repeated 3 times. After each wash, the plate was inverted on tissue paper to enhance moisture removal. Then, 1 mL of decreasing concentrations (96%, 70%, and 50%) of ethanol was added to the dish, swirled for 30 s, and discarded, followed by washing 5× with distilled water. Next, 1 mL of hematoxylin stain (Dako Agilent, CS70030-2, Santa Clara, CA, USA) was added to each dish and incubated for 10 min. The dishes were then washed 3× with tap water and incubated with 1 mL of tap water for 3 min. Once bluish color was enriched, dishes were washed 3× in distilled water. Subsequently, 1 mL eosin stain (Dako Agilent, CS70130-2) was added to the dishes and incubated for 10 min, followed by washing 2× with distilled water. Next, 2 mL of 100% ethanol was added to the dishes and stored at 4 °C. 

Images were captured using EVOS^®^ FL Auto microscope (Lifetechnologies, Carlsbad, CA, USA). CF population was determined at stromal regions surrounding the tumor cluster boundary as opposed to the NCF regions that were measured at 150 µm away from any tumor periphery. Cell count and eosin stain intensity was measured within defined areas placed randomly in the CF and NCF regions. A total of 50 regions were measured at the CF and NCF populations of the co-culture. Image J plugins: Cell Counter, Color Deconvolution (H&E vector; Color 2) were used. Color 2 versions representing the eosin stain were applied to produce scatter plots using plugin Interactive 3D Surface Plot v2.4.

In vivo staining: The experiment was performed by Lab Technician Anja Steinle. Briefly, tumor tissues were fixed in 4% PFA at 4 °C for 48 h. Tissues were paraffin-embedded and sectioned at a thickness of 3 µm. Tissue sections was mounted onto glass slides (VWR^®^ Superfrost^®^ Microscopic Slides, 564404, Radnor, PA, USA) and stored in an incubator overnight at 37 °C. The slides were deparaffinized in xylol and washed in decreasing concentrations of ethanol and, finally, distilled water. To demask the antigen structure, slides were placed into 1x target retrieval buffer (Dako Agilent, S2367842) of pH 9.0. The immersed slides were microwaved for 30 s and placed in a waterbath for 1 h at 97 °C, cooled for 20 min, and washed with distilled water for 5 min. Endogenous peroxidase was quenched with 5% methanol-based H_2_O_2_ solution for 15 min, followed by washing with distilled water for 5 min. Xenograft tissue sections were placed in 1× TBST buffer and stained with required concentration of primary antibody (IRF6: Atlas Antibodies, HPA 063121; FN1: Abcam, ab2413), diluted in 150 µL of antibody diluent (Dako Agilent, S302283-2), placed in a wet channel, and incubated overnight at 4 °C. The following day, slides were washed with 1x TBST buffer and incubated with secondary antibody (goat anti-rabbit-HRP conjugated: Dako Agilent, P0448), diluted (1:100) in 150 µL of antibody diluent for 35 min at 37 °C. Slides were washed with TBST buffer, and 150 µL DAB solution (Dako Agilent Kit, K3468) was added to the slides for 3 to 5 min, and tissue sections were placed in the dark. Slides were washed in normal water and distilled water and counterstained with hematoxylin (Dako Agilent, CS70030-2) for 10 min and washed. Subsequently, slides were washed in increasing concentrations of ethanol, and immersed in xylol for 5 min before being covered with mounting medium (VWR^®^ Entellan, 10911851, Radnor, PA, USA) and coverslip. IHC performed slides were imaged and analyzed using Image J and QuPath v0.4.3. Stromal vein width and fibroblast cell count were analyzed in 100 regions. With regard to necrotic zones, whole necrotic areas from three tissue sections in each xenograft were averaged and analyzed as a percentage with respect to the area of the whole tumor section. FN stain signal was analyzed in one tissue section per tumor. FN-stained areas were analyzed in seven regions across the tumor section per tumor. 

### 2.9. Protein Isolation and Western Blotting

BCSC + Fi co-cultures grown in 1:2 ratio was sorted for fibroblasts using FACS. Cell pellets obtained from control monocultures or co-cultures were lysed by resuspending in cOmplete^TM^ Protease Inhibitor Cocktail (Roche, 11697498001, Mannheim, Germany) and incubated for 30 min on ice. Cell debris was removed by centrifugation of the lysate at 13,000 rpm at 4 °C. DCTM Protein Assay Kit II (Bio-Rad Laboratories, 5000112, Hercules, CA, USA) was used to determine the protein concentration. Precast TGX gels (Bio-Rad Laboratories, 456-9036) was used in combination with Mini-PROTEAN^®^ Tetra Vertical Electrophoresis Cell chamber. Then, 15 µL of samples was loaded into each well. Gel was run at 130 V for 1.5–2 h and transferred to a PVDF membrane using the Trans-Blot Turbo Mini transfer packs (Bio-Rad laboratories, 1704156). Membrane was blocked with 5% BSA in 1x TBST for 1 h at room temperature. The membrane was incubated overnight with primary antibody (α-SMA: Antibodies, a82445; FN1: Antibodies, a89094; COL1A1: Thermo Fisher Scientific, PA5-29569; IRF6: Atlas Antibodies, HPA 063121; β-actin: Sigma Aldrich, A5441) that was diluted with 5% BSA in 1x TBST, on a rocking shaker at 4 °C. The following day, membrane was washed with 1x TBST and incubated with secondary antibody (Donkey anti-goat HRP: Thermo Fisher Scientific, A15999; Goat anti-rabbit IgG HRP: Agilent, P044801), diluted with 5% BSA in 1x TBST for 1 hr at room temperature. Membranes were subsequently washed and wetted with chemiluminescence reagent from Miracle-Star™ detection system (iNtRON Biotechnology, 16028, Gyeonggi-do, Korea). Protein bands were detected using Fusion SL detection system. Images were analyzed using Image J.

### 2.10. Orthotopic Breast Cancer Xenografts

In vivo experiments were performed in accordance with German Animal Welfare regulations and approved by the local authorities (animal protocol G13/114). Two control (BCSC1 + Scr Fi) mice and three mice per cell mixture: BCSC1 + KD Fi/ BCSC1 + OE Fi were utilized. NOD/SCID females (4–5 weeks old) were chosen and anesthetized using an isoflurane inhalator. A small sagittal incision (approx. 1.0 cm) was made on the shaved and sterilized abdomen that permits access to the mammary gland on both sides. Then, 1 × 10^5^ BCSC1 (EGFP) cells was mixed with 1 × 10^6^ non-irradiated fibroblasts (RFP) in 40 µL per gland of MSC medium and Matrigel (1:1 ratio). The seeding mixture was injected into the mammary fat pad on both sides of the animal. Each transplant was localized distal to the lymph node in the gland. Surgical incisions were sealed by suturing with a 5/0 thread. Animals were monitored twice per week for weight and tumor growth. Tumor volumes were measured using Vernier calipers and ultrasound. 

### 2.11. Statistical Analysis

Biological repeats are indicated as *n*-values. Data analysis was performed using GraphPad Prism 9. Datasets were compared using two-tailed, unpaired t test. Illustrated results were expressed as mean with SEM. Statistical significance of data was considered when *p*-value was less than 0.05. Symbols applied: (*)-*p* ≤ 0.05; (**)-*p* ≤ 0.01; (***)-*p* ≤ 0.001; (****)-*p* ≤ 0.0001. 

## 3. Results

### 3.1. Fibroblasts Densely Aggregate along the Periphery of BCSC Clusters

**Figure 1:**
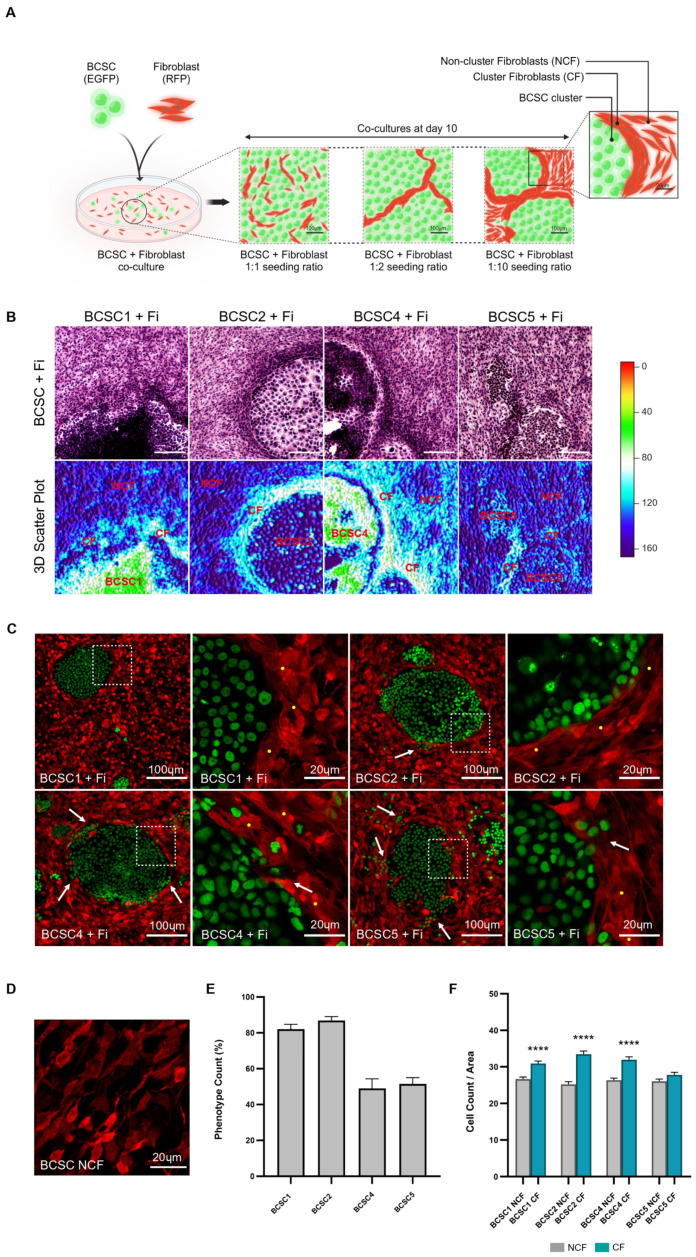
Fibroblasts aggregate and encapsulate BCSC clusters in co-culture. (**A**) Illustration of BCSC + fibroblast (BCSC + Fi) co-cultures at day 10, when seeded in 1:1, 1:2, and 1:10 ratios (BCSC: fibroblast). Magnified depiction of stromal cytoarchitecture in BCSC + Fi co-culture (1:10 ratio) showing aggregated cluster fibroblasts (CF) surrounding BCSC clusters and non-aggregated fibroblasts (NCF) situated away from BCSC clusters. (**B**) H&E-stained BCSC + Fi co-cultures (1:10 ratio, day 10) show fibroblast encapsulation at the periphery of BCSC clusters. Corresponding 3D scatter plot derived from eosin staining differentiates the high-intensity-stained CF population from the NCF population in co-cultures. Scale bar represents 100 µm. (**C**) Live-cell confocal images of BCSC + Fi co-cultures. White dotted squares represent a section of the CF (RFP) alignment along the BCSC (EGFP) cluster periphery. Yellow dots represent the closely knit fibroblasts that are circumferentially aligned in longitudinal fashion at the BCSC cluster periphery. White arrows indicate the disruption in the nematic order of fibroblasts at regions where BCSCs are disseminating from the cluster. (**D**) Representation of the disaggregated NCF population in co-culture. (**E**) Graphical representation of the measurement of SPE formation by the different BCSC lines, (*n* = 3). (**F**) Graphical representation of the increased cellular count in the CF populations when compared to the NCF populations in day 10 co-cultures. Statistical results are expressed as mean with SEM. Symbols applied: (****)-*p* ≤ 0.0001.

To investigate the interaction of BCSCs with stromal fibroblasts, a 2D in vitro co-culture model was developed. BCSCs and fibroblasts were seeded in varying ratios and the resulting multicellular organization was analyzed (Figure 1A). When the seeding density of fibroblasts was higher than that of BCSCs, fibroblast accumulation around the periphery of BCSC clusters was observed from day 5 to day 10 (Figure 1B and Figure S1A,B). Images at high magnification showed a dense stream of spindle-shaped fibroblasts that aggregated at the tumor–stromal interface to form a rigid encapsulation around the BCSC clusters as the co-culture matured (Figure 1C). This phenotype was referred to as the stromal phenotype of encapsulation (SPE). The aggregated fibroblasts were mostly aligned parallel to each other and their cellular morphology assumed a longitudinal orientation with respect to the peri-tumoral boundary. However, at positions where BCSC dissemination or cluster growth had occurred, the fibroblast alignment was disoriented and encapsulation was loosened.

Among the five BCSC lines established previously, namely, BCSC1, BCSC2, BCSC3, BCSC4, and BCSC5, clusters from four BCSC lines—BCSC1, BCSC2, BCSC4, and BCSC5—influenced this stromal phenotype of encapsulation (SPE) in co-cultured fibroblasts (Figure 1B and Figure S1A–C). The frequency of SPE was measured within a BCSC/fibroblast co-culture (BCSC + Fi), in relation to the total number of BCSC clusters (Figure 1E). BCSC1 + Fi and BCSC2 + Fi co-cultures showed a >80% occurrence of SPE when compared to BCSC4 + Fi and BCSC5 + Fi co-cultures that averaged at 40–60% in SPE formation. The decrease in phenotypic frequency could be partially attributed to the presence of disseminating clusters in BCSC4 and BCSC5 lines when compared to the predominant presence of tight clusters observed in BCSC1 and BCSC2 lines (Figure 1C). In order to closely investigate SPE and maintain consistency in results, BCSC1 and BCSC2 were used as the main cell lines in co-culture with fibroblasts as 2D models for the following in vitro experiments.

A seeding ratio of 1:10 (BCSC: fibroblast) in the BCSC + Fi co-culture model had proven the most efficient in mimicking tumor stem cell budding in surrounding stroma. The 1:10 ratio not only permitted the observation of the development of individual BCSC cells in an adequately populated fibroblastic environment but also enabled us to distinguish the SPE fibroblasts, henceforth referred to as cluster fibroblasts (CFs) in co-culture (Figure 1A,B and Figure S1A). Fibroblasts that were spatially distanced from the BCSC cluster vicinity, less aggregated, and did not participate in SPE formation around BCSC clusters were referred to as non-cluster fibroblasts (NCFs). The quantification of the nuclear count and eosin intensity in CF vs NCF in H&E-stained co-cultures confirmed the CF population to be denser when compared to the NCF population (Figure 1F and Figure S1D).

### 3.2. CF Population Creates an ECM-Rich Region around BCSC Clusters

**Figure 2:**
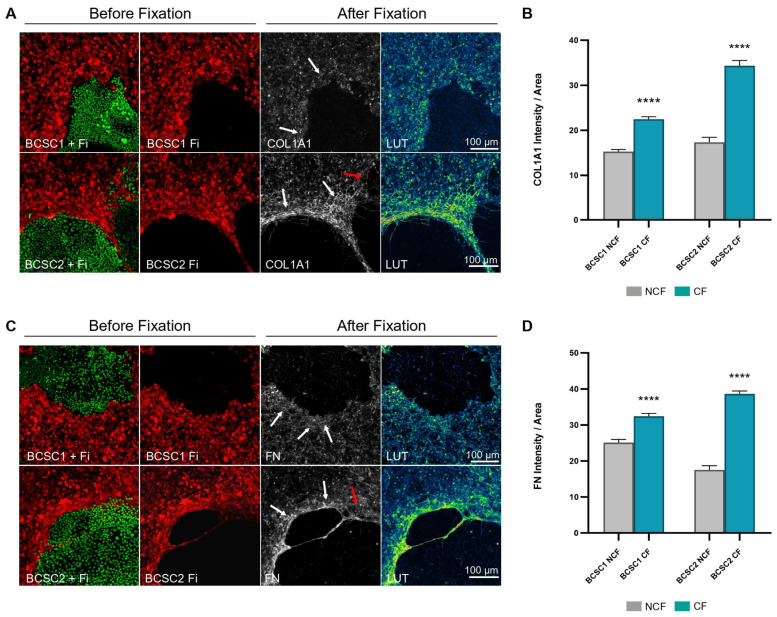
Accumulated ECM deposition overlaps with the CF population in the BCSC + Fi co-cultures. (**A**) Confocal images of day 10 BCSC + Fi co-cultures representing collagen type 1 (COL1A1)-rich deposition around BCSC clusters. (**B**) Bar graph represents the collagen intensity measurements at the CF and NCF populations of the co-cultures. White arrows indicate collagen fibers in close proximity to tumor cells. (**C**) Confocal images of day 10 BCSC + Fi co-cultures representing fibronectin (FN)-rich deposition at the BCSC cluster periphery. (**D**) Bar graph represents the fibronectin intensity measurements at the CF and NCF populations of the co-cultures. White arrows indicate fibronectin fibers in close proximity to tumor cells. Red arrows point to reduced ECM signal at areas of BCSC dissemination. Color gradient (LUT) is from blue (low-intensity signal) to yellow (high-intensity signal). (1:10 seeding ratio), BCSC (EGFP); Fibroblast (RFP). Statistical results are expressed as mean with SEM. *n* = 2, symbols applied: (****)-*p* ≤ 0.0001.

To assess stromal ECM in the BCSC + Fi co-culture model, 10-day co-cultures seeded in a 1:10 ratio were analyzed for the expression of type 1 collagen and fibronectin. Live-cell images of the co-cultures were taken before fixation to correlate ECM deposition with the fibroblast CF/NCF population. Confocal images of immunofluorescent stained co-cultures showed an enhanced deposition of type I collagen and fibronectin proteins at the periphery of the BCSC clusters (Figure 2A,C and Figure S2A,B). Comparison of heightened ECM localization with live-cell images indicated an overlapping of ECM accumulation with the CF population of the co-cultures. Areas of tumor dissemination into the CF-populated zones displayed reduced ECM in these regions.

The measurement of ECM at the CF and NCF populations of the co-cultures indicated a significant increase (*p* ≤ 0.0001) in collagen type 1 and fibronectin at the region of the CF population of the tumor–stroma interface (Figure 2B,D). However, observation of the stromal pattern of ECM distribution revealed differences in relation to the BCSC cell line used in co-cultures. BCSC1 + Fi co-cultures displayed an overall amplification in ECM at the CF and NCF populations with a slight increase in ECM at the tumor periphery. However, the BCSC2 + Fi co-cultures displayed a highly condensed ECM deposition around the BCSC2 cluster border forming an ECM ring at the tumor periphery, in contrast to a mild ECM presence in the stromal NCF area. Areas where BCSC cluster dissemination had occurred showed a reduced ECM presence.

Collagen and fibronectin fibers were also observed at regions surrounding BCSC clusters. ECM fibrils appeared wavy or linearized and were aligned parallel to the BCSC cluster border. The presence of stiff fibers was largely observed along the BCSC2 cluster periphery and, to a lesser extent, around BCSC1 clusters. 

To determine the observation of enhanced ECM to either be a product of CF accumulation or heightened CF ECM secretion at the BCSC cluster periphery, we analyzed the protein expression by western blotting. BCSC + Fi co-cultures seeded in a 1:10 ratio displayed a fibroblastic environment with a spectrum that ranges from a highly dense CF population at the tumor–stromal periphery to the less dense NCF population as the distance from the BCSC cluster boundary increases. Therefore, for western blot analysis, BCSC + Fi co-cultures were seeded in a ratio of 1:2 (BCSC: fibroblast) to eliminate the NCF population and to ensure all fibroblasts in the co-culture grew in close proximity with the BCSC clusters as the CF population (Figure S2C). Sorted BCSC CF from day 10 co-cultures were compared with control fibroblasts (Co Fi) that were harvested from fibroblast monocultures. BCSC1 CF and BCSC2 CF showed a reduced expression of collagen when compared to control fibroblasts (Figure S2D). However, the fibronectin expression in BCSC2 CF appeared much higher than in the control. The data indicates that BCSC CF’s association with accumulated ECM at the tumor–stromal boundary can be mostly concluded as a product of CF accumulation. However, the data also reveals the possibility that BCSC CF may have developed an enhanced ECM secretory capability specific to fibronectin.

Activated fibroblasts display enhanced ECM deposition and are identified by their expression of alpha smooth muscle actin (α-SMA) [31]. Western blot data of sorted BCSC CF displayed the presence of α-SMA. However, Co Fi had a higher expression of α-SMA when compared to the BCSC1 CF population and a relatively similar expression to BCSC2 CF population (Figure S2E). The results did not reveal a significant difference that was based on the BCSC-influenced activation of the CF population; therefore, α-SMA marker was not used to determine fibroblast activation.

### 3.3. Gene Expression Profiling of CF Population Reveals Upregulation of CAF Characteristics and Genes Regulating Fibrosis 

**Figure 3:**
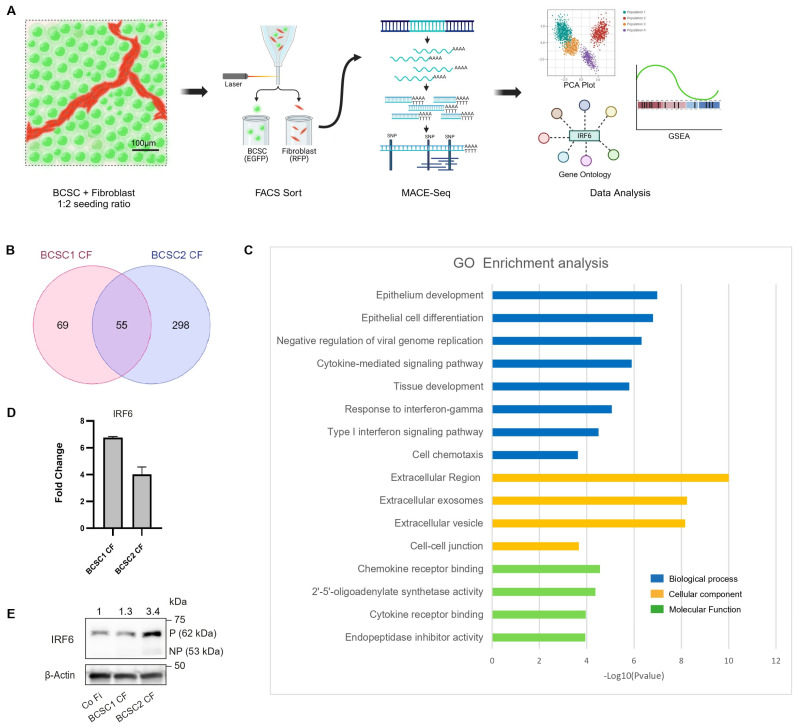
MACE-Seq analysis identifies IRF6 upregulation in BCSC CF populations. (**A**) Illustration depicting the sequence of procedures undertaken to analyze BCSC CF gene profile. (**B**) A common list of 55 genes were found to be significantly (*p* ≤ 0.01, FC ≥ 5) upregulated between the BCSC1 CF and the BCSC2 CF populations. (**C**) Gene Ontology (GO) enrichment analysis of the 55 gene list annotated in relation to biological processes, cellular localization, and molecular function. (**D**) Graphical representation of IRF6 mRNA expression in BCSC CF populations when compared to Co Fi (*n* = 2). (**E**) IRF6 protein expression in BCSC CF populations (*n* = 1).

The dense aggregation of CF and its corresponding ECM expression at the BCSC–stromal boundary led us to investigate the molecular signals in the CF population that influenced the SPE organization. CF population in BCSC + Fi co-cultures, seeded in a ratio of 1:2, were sorted from day 10 co-cultures and analyzed by MACE-sequencing (Figure 3A and Figure S3A). The gene expression profile of the BCSC1 CF and BCSC2 CF populations identified multiple DEGs when compared to control fibroblasts. Despite all BCSC lines being triple-negative, the principal component analysis (PCA) plot showed a certain variance amongst the samples of BCSC-influenced CF populations, indicating heterogeneity in BCSC lines and their corresponding CF populations (Figure S3B).

To analyze CAF characteristics in the BCSC CF populations, upregulated (*p* ≤ 0.01, FC ≥ 2) CAF genes were investigated. Analysis of CAF marker genes specific to TNBC identified the interleukin 7 receptor (IL7R) gene in both BCSC CF gene sets. In addition, complement component 3 (C3), C-X-C motif chemokine receptor 4 (CXCR4), and keratin 17 (KRT17) genes were specifically upregulated in BCSC1 CF populations. With regard to BCSC2 CF, C-X-C motif chemokine ligand 1 (CXCL1), and heat shock protein family A (HSP70), member 6 (HSPA6) genes were also identified [44]. BCSC1 CF also displayed the upregulation of common CAF marker microfibril-associated protein 5 (MFAP5) [45,46,47]. 

The additional characterization of CAF features in the BCSC CF gene profile revealed the upregulation of matrix metalloproteinases: MMP-3/9/11/13/15 in BCSC1 CF samples and MMP-7/15 in BCSC2 CF samples. The fibroblast activation and differentiation marker transforming growth factor beta 2 (TGF-ß2) was also upregulated in BCSC1 CF. CAF-related growth factor genes such as insulin-like growth factor 2 (IGF2) was observed in BCSC1 CF, whereas platelet-derived growth factor A (PDGFA) was expressed in BCSC2 CF [45]. CAF immunomodulatory function gene analysis identified commonly regulated C-X-C motif chemokine ligand 1 (CXCL1), C-X-C motif chemokine ligand 11 (CXCL11), C-C motif chemokine ligand 5 (CCL5), and C-C motif chemokine ligand 20 (CCL20) genes in both BCSC CF populations [48,49,50,51,52,53].

The pathway analysis of BCSC CF datasets using GSEA identified MYC targets v1 (*p* ≤ 0.0001) and oxidative phosphorylation (*p* ≤ 0.05) to be highly enriched in both BCSC1 CF and BCSC2 CF samples. In addition, BCSC2 CF also exhibited enrichment in E2F targets (ES: 0.63; *p* ≤ 0.0001), MYC targets v2 (ES: 0.56; *p* ≤ 0.0001), G2M checkpoint (ES: 0.56; *p* ≤ 0.0001), interferon alpha (IFN-α) response (ES: 0.53; *p* ≤ 0.0001), cholesterol homeostasis (ES: 0.49; *p* ≤ 0.0001), interferon gamma (IFN-γ) response (ES: 0.42; *p* ≤ 0.0001), mitotic spindle (ES: 0.41; *p* ≤ 0.0001), MTORC1 signaling (ES: 0.41; *p* ≤ 0.0001), spermatogenesis (ES: 0.43; *p* ≤ 0.001), estrogen response late (ES: 0.36; *p* ≤ 0.0001), peroxisome (ES: 0.38; *p* ≤ 0.02), estrogen response early (ES: 0.35; *p* ≤ 0.001), and glycolysis (ES: 0.34; *p* ≤ 0.004) (Figure S3 C). Several pathways that were enriched in the BCSC1 CF and BCSC2 CF populations were found to coincide with previously reported CAF mechanisms in breast, ovarian, and squamous cell carcinomas indicating the transition of BCSC CF population to a CAF phenotype [54,55,56,57].

To identify DEGs in the CF population that regulate the SPE formation, upregulated genes with a 5-fold change in expression and a significance smaller than 0.01 (*p* ≤ 0.01, FC ≥ 5) were used as cut-offs. A total of 55 genes were commonly upregulated among the two BCSC CF populations (Figure 3B, Table 1). Characterization of the 55 genes identified the genes to participate in several biological processes such as epithelial development (1.04 × 10^−7^), epithelial cell differentiation (1.57 × 10^−7^), negative regulation of viral genome replication (4.69 × 10^−7^), cytokine-mediated signaling pathway (1.17 × 10^−6^), tissue development (1.56 × 10^−6^), response to IFN-γ (8.69 × 10^−6^), type 1 interferon signaling pathway (3.11 × 10^−5^), and cell chemotaxis (2.34 × 10^−4^). With regard to the cellular component, the 55 genes were mostly localized to the extracellular region (1.00 × 10^−10^), extracellular vesicle (6.80 × 10^−9^), extracellular exosomes (5.70 × 10^−9^), and cell–cell junction (2.13 × 10^−4^). The molecular functions of the 55 genes were related to chemokine receptor binding (2.76 × 10^−5^), 2′-5′-oligoadenylate synthetase activity (4.21 × 10^−5^), cytokine receptor binding (1.08 × 10^−4^), and endopeptidase inhibitor activity (1.13 × 10^−4^) (Figure 3C).

The SPE formation by BCSC CF with the observation of increased ECM accumulation at the tumor–stromal periphery also led us to investigate fibrosis regulation in the significantly upregulated DEGs of the BCSC CF samples. Comparison of the 55-gene list with a fibrosis gene dataset (CTD Gene–Disease Association) revealed 38 genes in the list to correlate with the fibrosis process (Table 1).

The development of SPE formation by CF fibroblasts can be inferred as the consequence of several factors at play such as fibroblast activation, fibroblast recruitment, proliferation, changes in cell morphology, and the increase in cell–cell adhesion. Among the 38 fibrosis-regulating genes, the Interferon Regulatory Factor 6 (IRF6) gene was found to be annotated to several biological processes such as stress response, cell differentiation, cell cycle, anatomical structure development leading to cell morphogenesis, and cell adhesion (Figure S3D) [58]. Based on these findings, we hypothesized that BCSCs influence the upregulation of IRF6 transcription factor in fibroblasts, which may oversee several of the fibroblast characteristics observed in SPE formation pleiotropically.

IRF6 upregulation in the BCSC1 CF and BCSC2 CF populations at the RNA and protein level was confirmed (Figure 3D,E and Figure S3E,F). Western blot analysis revealed IRF6 protein to exist as phosphorylated (P) IRF6 (band: 62 kDa) and non-phosphorylated (NP) IRF6 (band: 50–53 kDa). IRF6 immunofluorescence in BCSC + Fi co-cultures (1:10) displayed the nuclear localization of IRF6 protein (Figure S3E). However, IRF6 overexpression was not restricted to the CF population alone and was also observed in the NCF population of the BCSC + Fi co-cultures.

### 3.4. IRF6 Knockdown Fibroblasts Fail to Aggregate at the BCSC Cluster Periphery

**Figure 4:**
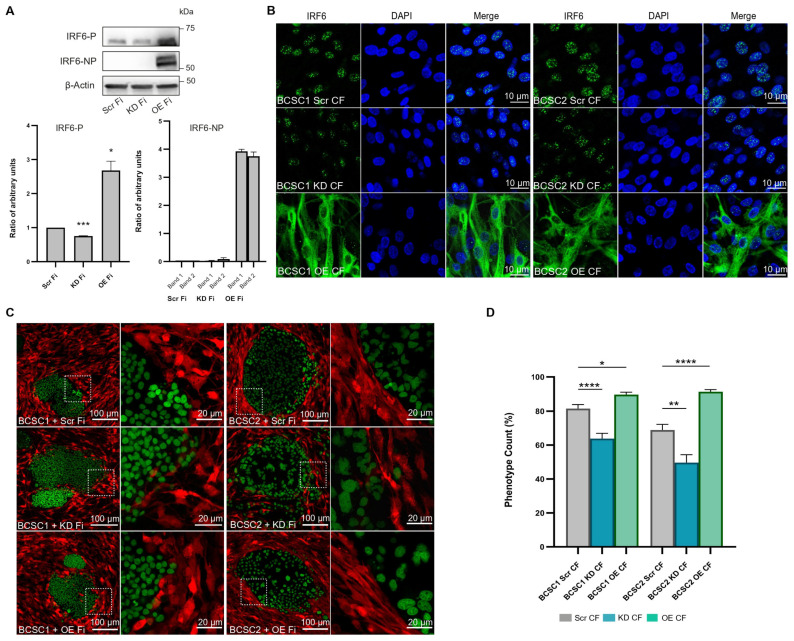
IRF6 KD fibroblasts show reduced SPE formation around BCSC clusters. (**A**) Western blot of IRF6 protein expression in Scr Fi, KD Fi, and OE Fi cultured without BCSCs (*n* = 2). (**B**) Immunofluorescent images of IRF6 protein expression in Scr CF (control scrambled cluster fibroblasts), KD CF (knockdown cluster fibroblasts), and OE CF (overexpression cluster fibroblasts) when co-cultured with BCSCs. (**C**) Live cell immunofluorescent images at day 10 of BCSC1 and BCSC2 co-cultures grown with Scr Fi/KD Fi/OE Fi. White dotted squares represent a section of the CF (RFP) alignment along the BCSC (EGFP) periphery that is magnified in the adjacent image. (**D**) Bar graph illustrating the measurement of SPE formation in BCSC Scr CF, BCSC KD CF, and BCSC OE CF (*n* = 2). (**B**–**D**): 1:10 seeding ratio. Symbols applied: (*)-*p* ≤ 0.05; (**)-*p* ≤ 0.01; (***)-*p* ≤ 0.001; (****)-*p* ≤ 0.0001.

To analyze the role of the IRF6 gene in SPE formation, IRF6 knockdown fibroblasts (KD Fi), IRF6 overexpression fibroblasts (OE Fi), and control fibroblasts (Scr Fi) were generated by viral transduction (Figure 4A). Protein quantification using immunofluorescent images showed BCSC1 KD CF and BCSC2 KD CF expressing roughly 40–50% less IRF6 compared to BCSC Scr CF (Figure 4B and Figure S4A). In contrast, OE CF expressed a high concentration of cytoplasmic IRF6 with a FC ≥ 5.5 when compared to BCSC Scr CF. BCSC CF was also analyzed by western blot to confirm IRF6 reduction (Figure S5C).

Analysis of the SPE phenotype in BCSC + KD Fi co-cultures revealed a reduction in the aggregation of the stromal fibroblasts around BCSC clusters. The BCSC KD CF appeared loosely assembled around the BCSC cluster boundary, showed reduced adherence amongst the fibroblast cells, and depicted a reduction in nematic alignment. In contrast, the BCSC OE CF displayed enhanced SPE formation with several rings of aggregated fibroblasts in parallel alignment, encapsulating the BCSC clusters (Figure 4C). The measurement of SPE formation indicated a significant decrease in BCSC1 KD CF (*p* ≤ 0.0001) and BCSC2 KD CF (*p* ≤ 0.01). By contrast, IRF6 overexpression resulted in a significant increase in SPE formation in BCSC1 OE CF (*p* ≤ 0.05) and BCSC2 OE CF (*p* ≤ 0.0001) (Figure 4D).

The appearance of loosely assembled, less-cohesive, single cells in BCSC KD CF prompted the investigation of the cellular morphology in transduced fibroblasts. KD Fi cultured without BCSCs displayed single cells with random cell orientation when compared to Scr Fi and OE Fi monocultures that exhibited closely adhered cells that were mostly aligned parallel to one another. In addition, the KD Fi appeared small and less elongated with short cell processes when compared to Scr Fi. The OE Fi displayed cells of a larger area with increased length and width (Figure S4B). This observation was validated with the quantification of the length-to-width ratio where the KD Fi displayed reduced elongation and were significantly smaller (*p* ≤ 0.0001) when compared to the control (Figure S4C). OE Fi was not quantified, as the mathematical formula did not accurately represent large cells with reduced length-to-width ratios. The cells were also measured for area, where IRF6 KD Fi showed a reduced cell area when compared to OE Fi; however, the difference was not significant (Figure S4D).

Previous research had associated IRF6 protein with cellular proliferation in keratinocytes; however, in fibroblasts, IRF6 was not observed to induce any difference [35,59,60]. Our data derived from growth curve analysis also did not reveal any difference with regard to IRF6 reduction or overexpression (Figure S4E).

### 3.5. BCSC KD CF Show Reduced Fibronectin Deposition

IRF6 was analyzed for its role in ECM deposition in the CF population of BCSC + Fi co-cultures. The observation of type 1 collagen deposition by KD CF around BCSC clusters indicated a less organized ECM distribution which mimicked the scattered KD CF phenotype (Figure S5A). Values derived from the protein quantification of collagen expression were found to be inconsistent with regard to KD CF and OE CF (Figure S5B,C).

**Figure 5:**
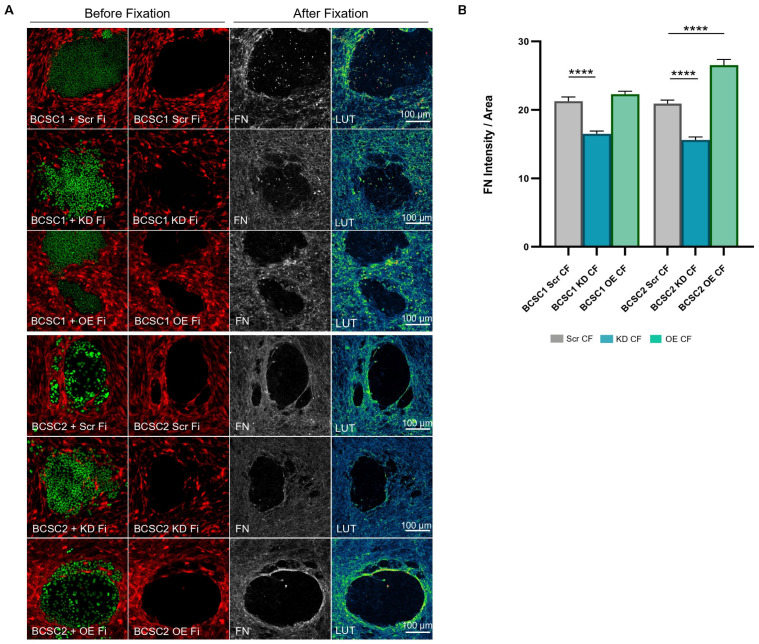
IRF6 expression positively regulates fibronectin deposition around BCSC clusters in 2D co-cultures. (**A**) Confocal images representing fibronectin deposition in BCSC + Scr Fi, BCSC + KD Fi, and BCSC + OE Fi co-cultures. Color gradient (LUT) is from blue (low-intensity signal) to yellow (high-intensity signal). (**B**) Bar graph represents the measurement of fibronectin expression by KD CF and OE CF in relation to Scr CF. (1:10 seeding ratio), BCSC (EGFP); Fibroblast (RFP). Statistical results are expressed as mean with SEM. Symbols applied: (****)-*p* ≤ 0.0001.

The observation of fibronectin protein deposition by KD CF around BCSC clusters displayed a reduced protein expression. In contrast, the OE CF showed a high fibronectin protein presence around the BCSC clusters (Figure 5A). Furthermore, the OE CF fibronectin matrix was highly condensed and formed an ECM ring around the BCSC2 clusters. The measurement of fibronectin deposition at the BCSC cluster periphery showed a significant decrease in fibronectin expression at the CF regions of BCSC + KD Fi co-cultures (*p* ≤ 0.0001) when compared to the BCSC Scr CF regions (Figure 5B). Conversely, the OE CF fibronectin surrounding BCSC1 clusters indicated values relatively similar to that of the control, whereas BCSC2 OE CF showed significantly higher (*p* ≤ 0.0001) fibronectin deposition than control. The Western blot analysis of fibronectin expression also showed a reduction in protein expression in the KD CF population when compared to Scr CF (Figure S5C). The experimental data indicate that IRF6 regulation in fibroblasts may be positively correlated with fibronectin expression in the setting of BCSC + Fi co-cultures.

### 3.6. IRF6 in Stromal Fibroblasts of Xenograft Models Regulate Stromal Development and Necrosis

**Figure 6:**
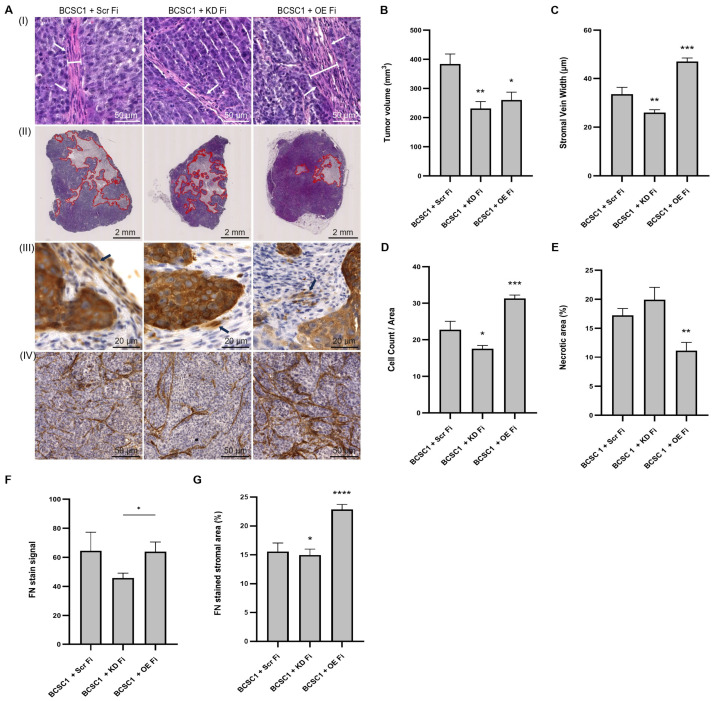
IRF6 in stromal fibroblasts enhances stromagenesis in BCSC xenografts. (**A**) (**I**) H&E-stained BCSC1 xenograft tumors grown with Scr Fi/KD Fi/OE Fi. Tumor sections were quantified for stromal vein width (white lines). White arrows indicate the longitudinal orientation of tumor fibroblasts that are parallelly aligned to adjacent fibroblasts at the tumor cell periphery. (**II**) Representation of necrotic area that is outlined in red. (**III**) IRF6-stained fibroblasts in tumor sections. Presence of nuclear and cytoplasmic IRF6 staining in all BCSC1 tumors. (**IV**) Images of fibronectin ECM stain in xenograft tumors sections. (**B**) Bar graph represents BCSC1 tumor volumes when grown with Scr Fi/KD Fi/OE Fi. (**C**) Bar graph illustrates reduced stromal vein width in BCSC1 tumors grown alongside KD Fi. (**D**) Representation of fibroblast count in stromal areas in BCSC1 xenograft tumor sections. (**E**) Representation of the percentage of necrotic area in BCSC1 tumors grown with Scr Fi/KD Fi/OE Fi. (**F**) Bar graph represents fibronectin (FN) stain intensity in tumor sections. (**G**) Representation of the percentage of FN-stained stromal areas in tumor sections. Symbols applied: (*)-*p* ≤ 0.05; (**)-*p* ≤ 0.01; (***)-*p* ≤ 0.001; (****)-*p* ≤ 0.0001.

The BCSC + Fi co-culture experiments had characterized fibroblast IRF6 function in a 2D TME setting. However, in order to assess the clinical significance of stromal fibroblast IRF6 activity in a tumor setting, in vivo experimentation was performed. The BCSC1 cell line was previously described to form orthotopic xenograft tumors with a reduced tumor onset period and rapid tumor growth kinetics (400 mm^3^ in 40 days) when compared to the BCSC2 cell line (400 mm^3^ in 75 days) [32]. Therefore, BCSC1 cell mixture with either Scr Fi or KD Fi or OE Fi was orthotopically transplanted into the mammary fat pad of immunodeficient mice. The animals were euthanized after 4 weeks and the tumor volumes were measured. The BCSC1 + Scr Fi tumors had volumes that averaged at 400 mm^3^, whereas the BCSC1 + KD Fi tumors displayed a significantly reduced tumor growth averaging at 250 mm^3^ (*p* ≤ 0.01). Interestingly, the BCSC1 + OE Fi tumor growth also displayed a significant reduction (*p* ≤ 0.05) in tumor volumes and the measurement was approximately 270 mm^3^ (Figure 6B).

Despite a BCSC/fibroblast seeding ratio of 1:10, the tumor tissue architecture resembled a 1:2 ratio of the BCSC + Fi 2D model. Therefore, all stromal fibroblasts in xenograft tumors were correlated with the CF population in co-cultures. The observation of tumor sections for fibroblast assembly at the tumor–stromal periphery relayed mostly parallelly aligned fibroblasts that assumed a longitudinal orientation with respect to the BCSC growth periphery (Figure 6A(I)).

Analysis of the tumor sections revealed two main features that varied with respect to BCSC tumors grown with either KD Fi or OE Fi: the stromal fraction and the necrotic area. The BCSC1 + OE Fi tumor sections displayed several expanded regions of fibroblast streams when compared to the BCSC1 + KD Fi tumor sections (Figure 6A(I)). The quantification of the stromal vein width in different regions of the tumors revealed the BCSC1 + KD Fi tumors to have a smaller (*p* ≤ 0.01) width of fibroblast veins that averaged at 25 µm when compared to the BCSC1 + Scr Fi tumors, whose stromal vein width averaged at 33 µm (Figure 6C). In contrast, the BCSC1 + OE Fi tumors displayed a significantly higher (*p* ≤ 0.001) width of fibroblast streams that averaged at 47 µm. In addition, the fibroblast number was also quantified in the stromal streams of tumor sections (Figure 6D). The BCSC1 + KD Fi tumors had a significantly lower (*p* ≤ 0.05) number of fibroblasts in a given stromal area when compared to the BCSC1 + OE Fi tumors that presented a higher (*p* ≤ 0.001) fibroblast count. The second prominent feature, the necrotic area in BCSC1 + KD Fi tumors, appeared to be more widespread when compared to the BCSC1 + OE Fi sections (Figure 6A(II)). The measurement of the area in the necrotic zones revealed an increase in necrosis in the BCSC1 + KD Fi tumors (Figure 6E). However, the BCSC1 + OE Fi tumors showed significantly reduced necrosis with *p* ≤ 0.01.

With regard to the IRF6 expression in the BCSC1 Fi of tumor tissue, only a minimal number of fibroblasts in the tissue sections were stained with IRF6 irrespective of BCSC1 tumors grown with Scr Fi, KD Fi, or OE Fi (Figure 6A(III)). The IRF6 expression in stromal fibroblasts of tumor tissues was not restricted to the nuclei as observed in the BCSC Fi of 2D models, but was also present in the cytoplasm in 3D xenograft tissues and were mostly found along the periphery of the tumor.

Based on the differences observed in fibronectin expression by BCSC CF in 2D experiments when co-cultured with KD Fi and OE Fi, BCSC1 tumor sections were also analyzed for fibronectin signal (Figure 6A(IV)). The quantification of fibronectin intensity in the stromal areas revealed a decrease (*p* ≤ 0.05) in expression in BCSC1 + KD Fi tumor tissues when compared with BCSC1 + OE Fi tumor tissues (Figure 6F). However, similar to BCSC1 + OE Fi co-cultures, the BCSC1 + OE Fi xenografts also showed no significant increase in fibronectin expression and displayed a value that was comparable to the staining in control tumors. Yet, a closer observation of the fibronectin-stained tumor sections revealed a greater area of fibronectin staining in the stroma of BCSC1 + OE Fi tumors when compared to BCSC1 + KD Fi tumors (Figure 6G). The measurement of the area fraction stained by stromal fibronectin in BCSC1 + KD Fi xenografts revealed a slight but significant reduction (*p* ≤ 0.05) in stromal area that averaged at 14% when compared to BCSC1 + Scr Fi tumor sections that displayed an area of 16%. In contrast, the BCSC1 + OE Fi tumors expressed higher stromal area fractions that averaged at 22.5% with a significance of *p* ≤ 0.0001.

The results from the above experiments suggest that in a tumor setting, the IRF6 expression in fibroblasts increases the stromal area in the BCSC niche and provides survival signals to BCSC1 cells that guards the cells against necrosis. Similarly, in another experiment, the orthotopic transplantation of a cell mixture of BCSCs and wild-type fibroblasts, and, conversely, only wild-type fibroblasts were performed and observation was made at Day 8 and Day 11 from transplantation. Data from the experiment showed a complementary result, where BCSCs ensured the survival of human fibroblasts, whereas, when fibroblasts were transplanted without BCSCs, the transplant did not survive for an extended period of time (Figure S6). Taken together, these results indicate a reciprocal relationship that is shared between the BCSCs and fibroblasts to ensure survival.

## 4. Discussion

The reactive stroma of the breast TME is abundantly populated with activated fibroblasts [61,62]. Tissue organization data from previous studies indicate the accumulation of encircling fibroblasts around tumor cell nests to be a common feature in breast cancer [22,24,25]. Similarly, our model of the BCSC microenvironment in 2D co-culture with a seeding ratio of 1:10 (BCSC: fibroblast) also depicted the accumulation of cluster fibroblasts along the BCSC cluster periphery. The CF population possessed an elongated/stretched morphology that mostly adhered to each other in nematic order to form a rigid multicellular encapsulation along the BCSC–stromal boundary. The longitudinal orientation of fibroblasts at the expanding BCSC periphery can be reasoned with the observation from an earlier study that reported the orientation of stretched fibroblast structures to align perpendicular to the direction of mechanical stress [63].

The second major characteristic of CAFs and a hallmark of cancer is the presence of abnormal ECM alterations in the TME [64,65]. The stroma in breast cancers is characterized by a large deposition of ECM, mainly collagen type I and fibronectin proteins which are observed in areas of dense connective tissue stroma that are in close proximity to tumor cell nests [26,28,66]. Similarly, the BCSC-influenced CF phenotype also displayed ECM modifications with the presence of enhanced collagen type 1 and fibronectin proteins at the BCSC periphery. The presence of parallelly aligned wavy fibers and, to a lesser extent, linearized ECM fibers surrounding the BCSC cluster periphery suggest that the BCSC’s influence on the CF population results in the formation of TACS-1 and TACS-2 signatures that has been previously described to signify premalignant, quiescent tumors with signs of growth [67]. However, at sites of BCSC dissemination, the perpendicularly aligned TACS-3 signature was not observed due to an absence of ECM expression in these regions. This could be explained as the result of the activity of various MMPs that could be secreted by the CF population which could degrade the deposited ECM to create space for the disseminating cancer cells in a 2D setting [68].

With regard to the gene expression profile, it was logical to discover upregulated DEGs of the BCSC CF population to correlate with the TNBC CAF marker genes [44]. Additionally, commonly expressed CAF genes such as *MMP*s, *CXCL1*, and *IGF2* were also expressed by BCSC CF, which suggests that BCSCs influence the transition of the CF population to a CAF phenotype [45]. Also, several enriched pathways in BCSC CF were found to overlap with hallmark pathways expressed by breast cancer CAF subtypes which further implies the transformation of BCSC CF to CAFs [56]. Data from GSEA reveal the BCSC CF populations to commonly express the enrichment of genes in MYC targets and oxidative phosphorylation, including glycolysis in BCSC2 CF populations. Coupled with the BCSC CF GO data that correlates the spatial enrichment of DEGs with extracellular vesicles and exosomes, it can be inferred that CF populations may be programmed to secrete nutrient-rich metabolites for BCSC growth [69]. Additionally, ECM stiffness has also been implicated in the increase in glycolysis and oxidative phosphorylation pathways in CAFs [57]. Analogously, the observation of a dense ECM around BCSC clusters in our co-culture models may also function to promote the increase in the transfer of metabolic cargo from the CF population for BCSC survival. With regard to BCSC2 CF, additional pathways were found to be enriched, such as cell-cycle-related E2F targets and G2M checkpoint signaling that correlates with hyper-proliferative CAFs in breast cancer lesions and ovarian carcinomas [54,70]. Based on the above data, BCSC CF can be implicated in the regulation of core CAF pathways such as fibroblast activation, energy transfer, and matrix remodeling that together are capable of sustaining tumorigenesis. However, additional experimentation is required to validate the downstream functions of the enriched BCSC CAF pathways in the context of a BCSC niche.

Investigation of tumor fibrosis mechanisms in BCSC CF revealed two-thirds of the highly expressed 55 genes to be implicated in fibrosis regulation. Based on the hypothesis that a composition of several factors may function together to give rise to SPE formation, transcription factor IRF6 was chosen for analysis. Investigation of the fibrosis-contributing *IRF6* gene in BCSC CF revealed the upregulation of IRF6 protein with the presence of two bands: IRF6-P and IRF6-NP. Under resting conditions, interferon regulatory factors (IRFs) have been found to accumulate in the cytoplasm. Upon the induction of stimuli, IRFs become phosphorylated and active, and are translocated to the nucleus [71,72]. However, in contrast to its family members, IRF6-P is not immediately detected in the nucleus and is also found in the cytoplasm [73]. Due to the ambiguity surrounding the activated state of IRF6-P and the lack of literature characterizing IRF6 at the molecular level in fibroblasts, IRF6 protein in BCSC Fi was not distinguished in terms of phosphorylation [74].

In 2D co-culture, the IRF6 KD CF morphology presenting single cells with reduced aggregation and polarity correlates with previous observations on IRF6-altered keratinocytes and fibroblasts [35,75]. IRF6 has been associated with targeting cell adhesion, which may explain the scattered appearance of the IRF6 KD Fi and BCSC KD CF population [35,58]. In addition, BCSC KD CF also exhibited reduced cell elongation with random cell orientation that disordered the nematic stream observed around BCSC clusters. A previous study suggested that fibroblast attachment to collagen fibers induces mechanical stimulation that changes the morphology of the cell to an elongated phenotype [76,77]. This observation may also prove true in the case of IRF6 KD CF; however, further experimentation is required to confirm this possibility. In contrast, the BCSC OE CF population displayed a compact aggregate of elongated fibroblast cells that were structurally organized in nematic alignment, which reflects the stretched fibroblast phenotype observed in CAFs [78]. Therefore, our data not only validates the IRF6 association with the formation of the SPE phenotype but also suggests that IRF6 may play an intermediary role in mechanotransduction that stimulates the formation of an elongated CAF structure.

Similar to the observation of an enhanced SPE phenotype in 2D BCSC co-cultures with OE Fi, BCSC1 xenograft tumors grown with OE Fi also displayed an increased stromal area and fibroblast density. In contrast, BCSC1 tumors cultured with KD Fi showed reduced stromal width with a decreased fibroblast cell count, suggesting the ability of IRF6 to induce stromagenesis in the BCSC niche [79]. Based on the observation of an accumulation of OE CF around BCSC clusters and stromagenesis in BCSC xenografts, it could also suggest that IRF6 expression in fibroblasts may promote the spatial proximity of fibroblast cells with tumor cell clusters. The data from our experiments also link the IRF6 expression in activated stromal fibroblasts to BCSC cell survival and a reduction in necrosis, which is similar to a previous study that observed the depletion of FAP^+^ CAFs in the TME to be associated with necrosis in Lewis lung carcinoma mouse models [80]. However, considering the observation of a minimal number of IRF6-expressing fibroblasts present in all BCSC1 tumors, it can be proposed that the signals relayed by IRF6 expression in stromal fibroblasts may possibly be an early event in the development of BCSC xenografts. Furthermore, our data also correlate fibroblast IRF6 function with fibronectin expression in 2D co-cultures and expanded fibronectin regions in the TME. These data suggest that the pleiotropic function of the IRF6 transcription factor may also oversee ECM remodeling in the BCSC niche. It is important to note that despite the observation of stromagenesis and reduced necrosis in BCSC1 + OE Fi tumors, it is unclear why the tumors display reduced growth, especially when survival is enhanced. Further investigation will be needed to interpret whether the reciprocal effects of IRF6-expressing stromal fibroblasts reduce apoptosis and influence quiescence in the BCSC population.

The SPE formation by stromal fibroblasts observed in the BCSC + Fi co-cultures is a common occurrence in multiple cancers [22,24,81]. Data from our in vitro co-culture model suggest that the BCSC-influenced IRF6 upregulation in CAFs is associated with fibroblast encapsulation around tumor clusters. In BCSC xenografts, the difference in IRF6 expression shows a clear variation in the development of the stroma and its corresponding influence of survival on the BCSC cells in the tumor niche. Therefore, we propose IRF6 in stromal fibroblasts to be a significant candidate for further investigation into the mechanism of tumor progression from the CSC niche and, perhaps, also, in the TME.

## 5. Conclusions

In this study, we characterized the development of a BCSC niche in relation to fibroblasts. Our data demonstrate that fibroblasts accumulate in the vicinity of triple-negative BCSCs, and are influenced by the CSC to transition to a CAF phenotype and induce fibrosis at the epithelial–stromal boundary. The upregulated IRF6 gene expression influenced by the BCSCs in surrounding stromal fibroblasts was found to participate in tumor fibrosis by regulating fibroblast elongation, cellular aggregation and ECM remodeling at the tumor periphery in 2D co-cultures. In 3D BCSC xenografts, IRF6 expression in tumor-associated stromal fibroblasts induced stromagenesis in combination with decreased necrotic growth. The maintenance of a BCSC niche depends on factors that promote stemness and cell survival, which is provisioned by the stromal support in the surrounding region. Based on our findings, we propose that the stromal IRF6 participation in TME development is a critical factor in the maintenance of the BCSC niche.

## Figures and Tables

**Table 1:** List of 55 genes that are commonly upregulated in the BCSC1 CF and BCSC2 CF samples.

No.	Gene	Description	BCSC1 CF (FC)	BCSC1 CF (*p*-Value)	BCSC2 CF (FC)	BCSC2 CF (*p*-Value)
1.	AP1M2	Adaptor Related Protein Complex 1 Subunit Mu 2	6.4	2.91 × 10^−5^	10.7	2.08 × 10^−14^
2.	BST2	Bone Marrow Stromal Cell Antigen 2	8.4	3.85 × 10^−11^	10.2	3.17 × 10^−16^
3.	C6orf223	Long Intergenic Non-Protein Coding RNA 3040	5.9	4.90 × 10^−8^	8.5	2.06 × 10^−16^
4.	CA2	Carbonic Anhydrase 2	7.7	1.73 × 10^−7^	9.8	3.47 × 10^−12^
5.	CBLC	CBL Proto-Oncogene C	7.4	6.48 × 10^−6^	7.7	9.73 × 10^−7^
6.	CCL5	C-C Motif Chemokine Ligand 5	5.9	4.17 × 10^−5^	7.4	6.59 × 10^−8^
7.	CCL20	C-C Motif Chemokine Ligand 20	6.2	1.36 × 10^−5^	6.1	1.28 × 10^−5^
8.	CD70	Cluster of Differentiation 70	6.7	1.35 × 10^−5^	7.9	1.24 × 10^−7^
9.	COBL	Cordon-Bleu WH2 Repeat Protein	6.3	1.29 × 10^−4^	9.0	4.00 × 10^−9^
10.	CPVL	Carboxypeptidase Vitellogenic Like	5.4	8.33 × 10^−4^	9.1	6.53 × 10^−10^
11.	CRB3	Crumbs Cell Polarity Complex Component	7.3	1.92 × 10^−6^	8.9	1.77 × 10^−9^
12.	CXCL11	C-X-C Motif Chemokine Ligand 11	6.7	2.97 × 10^−6^	6.2	1.21 × 10^−5^
13.	DEPP1	DEPP Autophagy Regulator 1	6.7	3.05 × 10^−5^	10.5	2.97 × 10^−12^
14.	DSC3	Desmocollin 3	6.4	3.05 × 10^−5^	9.3	1.35 × 10^−10^
15.	ELF3	E74 Like ETS Transcription Factor 3	8.5	1.05 × 10^−13^	11.4	1.33 × 10^−24^
16.	EPPK1	Epiplakin	7.2	1.11 × 10^−5^	7.5	2.39 × 10^−6^
17.	FGFBP1	Fibroblast Growth Factor Binding Protein 1	8.1	2.36 × 10^−6^	9.9	1.89 × 10^−9^
18.	FOXA1	Forkhead box A1	6.1	1.35 × 10^−4^	9.4	1.47 × 10^−10^
19.	FXYD3	FXYD Domain Containing Ion Transport Regulator 3	9.4	9.70 × 10^−10^	9.5	9.85 × 10^−10^
20.	GJB2	Gap Junction Protein Beta 2	6.7	1.35 × 10^−5^	9.5	7.78 × 10^−11^
21.	GRHL2	Grainyhead Like Transcription Factor 2	6.8	2.21 × 10^−5^	9.3	7.91 × 10^−10^
22.	IGSF3	Immunoglobulin Superfamily Member 3	6.7	7.13 × 10^−5^	8.9	2.57 × 10^−8^
23.	IQANK1	IQ Motif and Ankyrin Repeat Containing 1	6.1	8.73 × 10^−5^	8.7	2.41 × 10^−9^
24.	IRF6	Interferon Regulatory Factor 6	7.0	3.40 × 10^−12^	6.2	1.32 × 10^−9^
25.	KDF1	Keratinocyte Differentiation Factor 1	6.8	1.04 × 10^−5^	9.7	2.44 × 10^−11^
26.	KRT7	Keratin 7	5.6	1.00 × 10^−12^	7.9	3.37 × 10^−25^
27.	LAD1	Ladinin 1	8.8	2.14 × 10^−8^	10.2	3.78 × 10^−11^
28.	LCN2	Lipocalin 2	8.8	8.42 × 10^−9^	13.2	4.43 × 10^−19^
29.	LTB	Lymphotoxin Beta	6.1	9.54 × 10^−5^	9.8	1.35 × 10^−11^
30.	MIR205HG	MIR205 host gene	9.9	1.39 × 10^−10^	6.8	2.35 × 10^−5^
31.	MISP	Mitotic Spindle Positioning	6.7	5.26 × 10^−5^	8.0	2.91 × 10^−7^
32.	MPZL2	Myelin Protein Zero Like 2	6.9	1.55 × 10^−5^	9.5	2.39 × 10^−10^
33.	MST1R	Macrophage Stimulating 1 Receptor	5.9	1.71 × 10^−4^	8.5	6.05 × 10^−9^
34.	NECTIN4	Nectin Cell Adhesion Molecule 4	5.7	3.82 × 10^−5^	7.5	9.49 × 10^−9^
35.	NMU	Neuromedin U	7.5	2.97 × 10^−6^	7.5	2.46 × 10^−6^
36.	OAS1	2′-5′-Oligoadenylate Synthase 1	9.8	2.04 × 10^−16^	7.9	1.29 × 10^−10^
37.	OASL	2′-5′-Oligoadenylate Synthase Like	6.8	9.78 × 10^−9^	6.9	4.08 × 10^−9^
38.	PI3	Peptidase Inhibitor 3	6.0	2.48 × 10^−11^	5.1	2.49 × 10^−8^
39.	PROSER2	Proline And Serine Rich 2	6.5	5.60 × 10^−5^	7.4	2.49 × 10^−6^
40.	PRSS8	Serine Protease 8	6.2	2.51 × 10^−4^	9.6	7.05 × 10^−10^
41.	RAB25	Member RAS Oncogene Family	7.6	4.76 × 10^−5^	10.5	2.52 × 10^−9^
42.	S100A14	S100 Calcium Binding Protein A14	7.7	2.76 × 10^−11^	10.0	1.12 × 10^−18^
43.	S100A9	S100 Calcium Binding Protein A9	7.2	1.15 × 10^−6^	10.0	9.77 × 10^−13^
44.	SAA2	Serum Amyloid A2	6.7	2.09 × 10^−5^	8.45	1.55 × 10^−8^
45.	SCNN1A	Sodium Channel Epithelial 1 Subunit Alpha	7.0	1.64 × 10^−7^	7.3	3.46 × 10^−8^
46.	SFN	Stratifin	8.4	1.15 × 10^−9^	9.5	3.75 × 10^−12^
47.	SLPI	Secretory Leucocyte Peptidase Inhibitor	7.7	7.67 × 10^−7^	11.1	1.17 × 10^−13^
48.	SMOC1	SPARC Related Modular Calcium Binding 1	7.6	5.85 × 10^−7^	9.0	1.07 × 10^−9^
49.	SNX10	Sorting Nexin 10	6.8	1.96 × 10^−5^	6.7	1.53 × 10^−5^
50.	SPINT2	Serine Peptidase Inhibitor, Kunitz Type 2	5.4	1.06 × 10^−11^	8.5	2.45 × 10^−28^
51.	TACSTD2	Tumor Associated Calcium Signal Transducer 2	6.2	4.29 × 10^−12^	8.5	2.06 × 10^−22^
52.	TGFA	Transforming Growth Factor Alpha	7.0	4.50 × 10^−6^	9.6	2.93 × 10^−11^
53.	TINAGL1	Tubulointerstitial Nephritis Antigen Like 1	9.8	4.63 × 10^−11^	10.8	2.77 × 10^−13^
54.	WFDC2	WAP Four-Disulphide Core Domain 2	6.1	5.80 × 10^−5^	6.3	1.81 × 10^−5^
55.	XIST	X Inactive Specific Transcript	6.2	1.36 × 10^−4^	8.3	4.68 × 10^−8^

Significantly upregulated (*p* ≤ 0.01, FC ≥ 5) 55 genes in high SPE forming BCSC1 CF and BCSC2 CF samples. Fibrosis-regulating 38 genes are highlighted.

## Data Availability

The data that support the findings of this study are available from the corresponding author upon reasonable request.

## Supplementary Materials

The following supporting information can be downloaded at: https://www.mdpi.com/article/10.3390/cells13171466/s1, Figure S1: Fibroblasts encapsulate BCSC clusters in co-culture; Figure S2: ECM expression in BCSC + Fi co-cultures and seeding ratio 1:2; Figure S3: MACE-Seq analysis: FACS sort, PCA plot, GSEA plots, IRF6 gene annotation, and protein expression; Figure S4: IRF6 protein quantification, IRF6 associated fibroblast morphology, growth curve analysis; Figure S5: COL1A1 ECM expression in BCSC + Scr Fi, BCSC + KD Fi and BCSC + OE Fi co-cultures, ECM expression analysis using western blot; Figure S6: BCSC1 tumors at Day 8 and Day 11 compared to control.

## Abbreviations

BCSC	Breast cancer stem cells
Fi	Fibroblast
BCSC + Fi	BCSC/fibroblast co-culture
CAF	Cancer-associated fibroblasts
CSC	Cancer stem cells
COL1A1	Collagen type I alpha 1 chain
ECM	Extracellular matrix
FN	Fibronectin
DEG	Differentially expressed gene
GO	Gene ontology
IRF6	Interferon regulatory factor 6
CF	Cluster fibroblasts
NCF	Non-cluster fibroblasts
Co Fi	Control fibroblasts
KD Fi	Knockdown fibroblasts
OE Fi	Overexpression fibroblasts
Scr CF	Scrambled cluster fibroblasts
KD CF	Knockdown cluster fibroblasts
OE CF	Overexpression cluster fibroblasts
SPE	Stromal phenotype of encapsulation
TACS	Tumor-associated collagen signatures
TME	Tumor microenvironment
TNBC	Triple-negative breast cancer
