# Supplementary material for: Breast Cancer Stem Cells Upregulate IRF6 in Stromal Fibroblasts to Induce Stromagenesis
Source: Cells. 2024 Aug 31;13(17):1466. doi: 10.3390/cells13171466 (PMC11393902; doi:10.3390/cells13171466)
Supplement: Supplementary file 1 [file cells-13-01466-s001.zip › cells-3106680-supplementary.pdf]

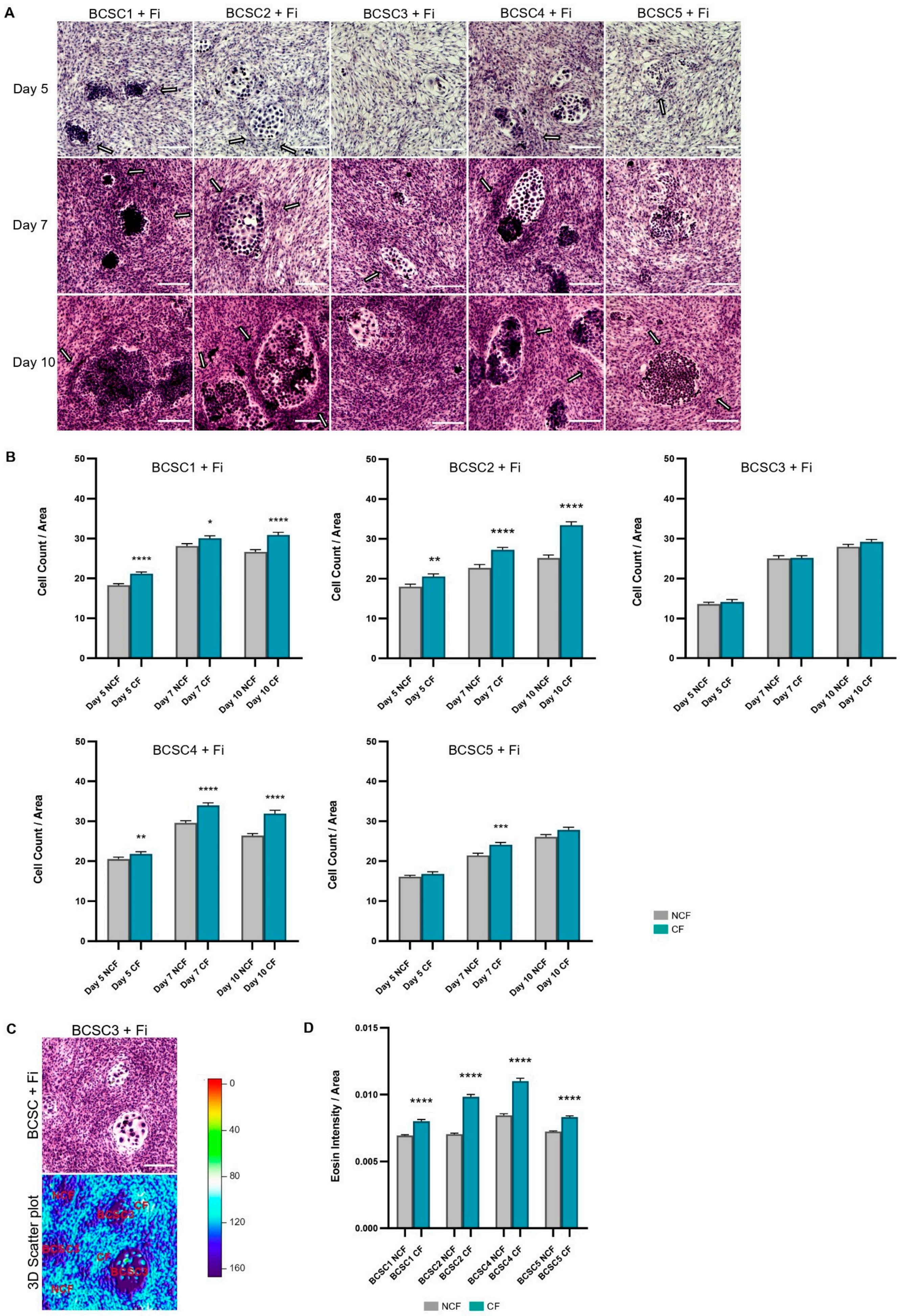
A
BCSC1 + Fi
BCSC2 + Fi
BCSC3 + Fi
BCSC4 + Fi
BCSC5 + Fi
Day 5
Day 7
Day 10
B
BCSC1 + Fi
BCSC2 + Fi
BCSC3 + Fi
BCSC4 + Fi
BCSC5 + Fi
Cell Count / Area
0
10
20
30
40
50
Day 5 NCF
Day 5 CF
Day 7 NCF
Day 7 CF
Day 10 NCF
Day 10 CF
****
*
**
***
NCF
CF
C
BCSC3 + Fi
BCSC + Fi
3D Scatter plot
NCF
CF
BCSC3
0
40
80
120
160
D
Eosin Intensity / Area
0.000
0.005
0.010
0.015
BCSC1 NCF
BCSC1 CF
BCSC2 NCF
BCSC2 CF
BCSC4 NCF
BCSC4 CF
BCSC5 NCF
BCSC5 CF
****
NCF
CF

Figure S1:

(A) H and E stained BCSC + Fi co-cultures in 1:10 ratio, fixed at day 5, day 7 and day 10. Arrows indicate the accumulation of fibroblasts at the BCSC cluster periphery. Scale bar represents 100µm. **(B)** Bar graphs representing the nuclear count of the CF (cluster fibroblasts) and NCF (non-cluster fibroblasts) populations for each of the 5 BCSC + Fi co-cultures. Fibroblast population was quantified at the cancer-stromal interface (CF) and 150µm away from the tumor periphery (NCF). **(C)** BCSC3 + Fi co-cultures at day 10 stained with H and E and its representative 3D scatter plot. Scale bar represents 100µm. **(D)** Bar graph representing the eosin intensity of the CF and NCF populations in BCSC + Fi co-cultures.

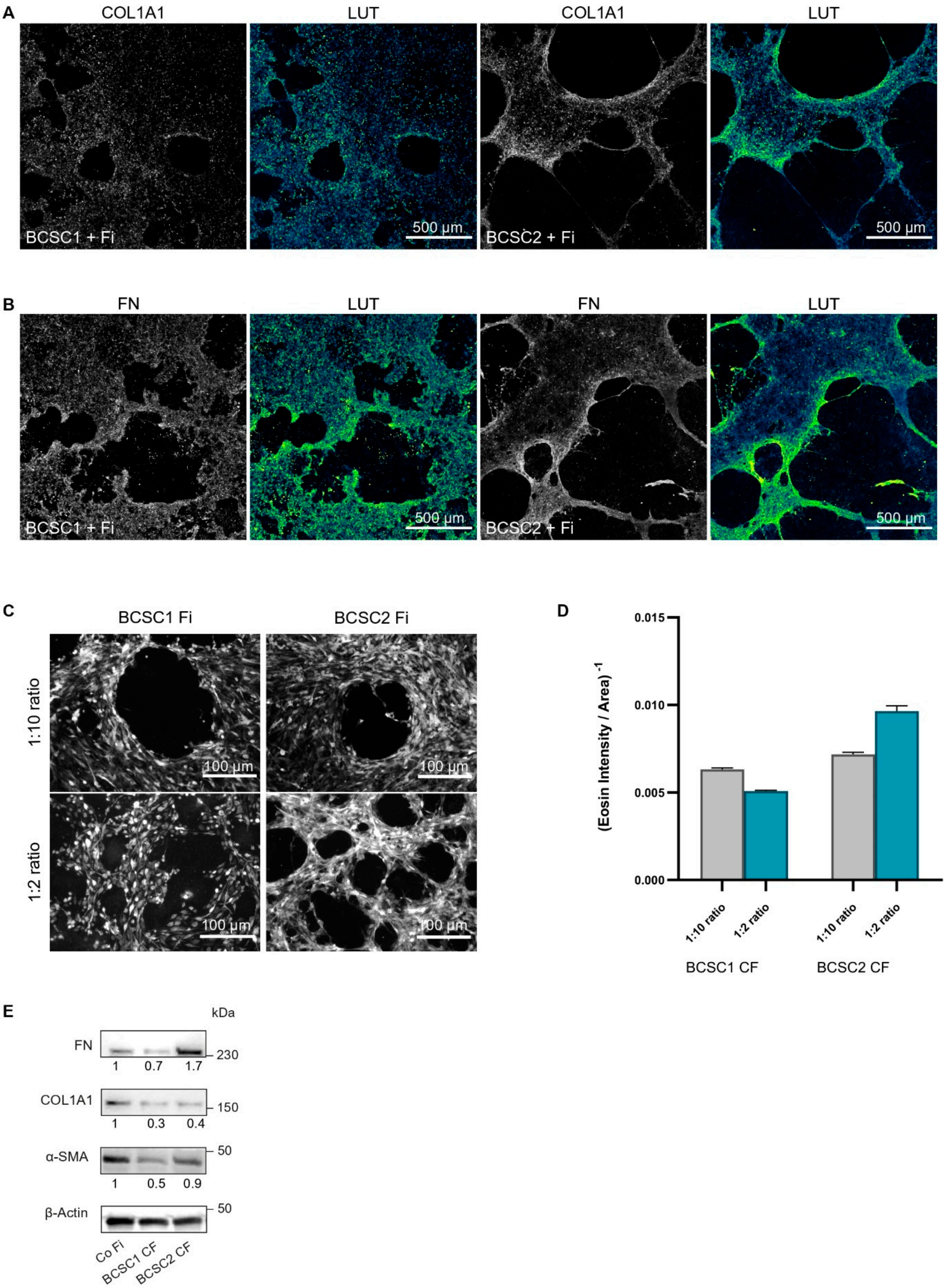

Figure S2:

(A) Tile scan images representing collagen type 1 (COL1A1) distribution in BCSC + Fi co-cultures (1:10 ratio). **(B)** Tile scan images representing fibronectin (FN) distribution in BCSC + Fi co-cultures (1:10 ratio). **(C)** Confocal images of BCSC1 Fi and BCSC2 Fi at day 10, seeded in 1:10 and 1:2 ratios (BCSC: fibroblast). **(D)** Graphical representation of eosin intensity measurement in 1:10 ratio compared to 1:2 ratio in BCSC1 CF and BCSC2 CF. **(E)** Western Blot analysis of COL1A1, FN, and α-SMA expression in BCSC1 CF and BCSC2 CF when compared to control fibroblasts (Co Fi). As α-SMA and β-actin share the same molecular weight, β-actin was run on a different gel (n=1). Colour gradient (LUT) is from blue (low intensity signal) to yellow (high intensity signal).

A

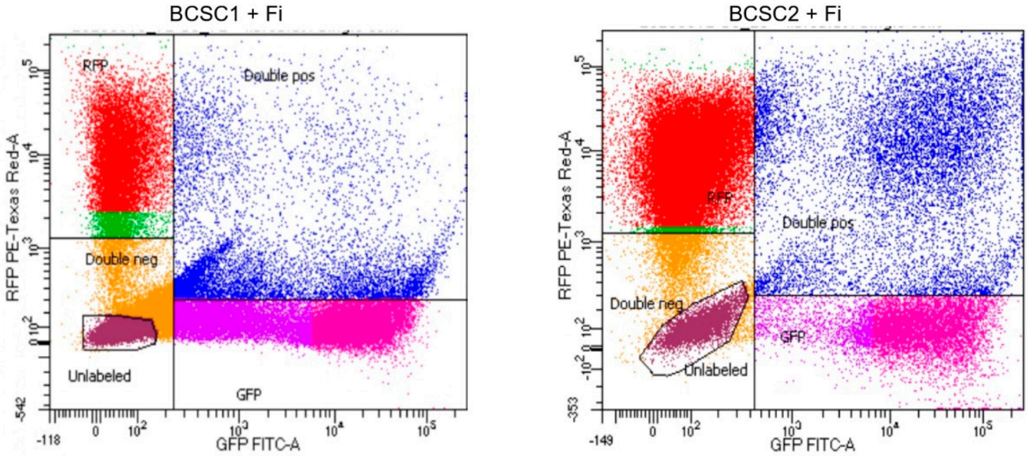

B

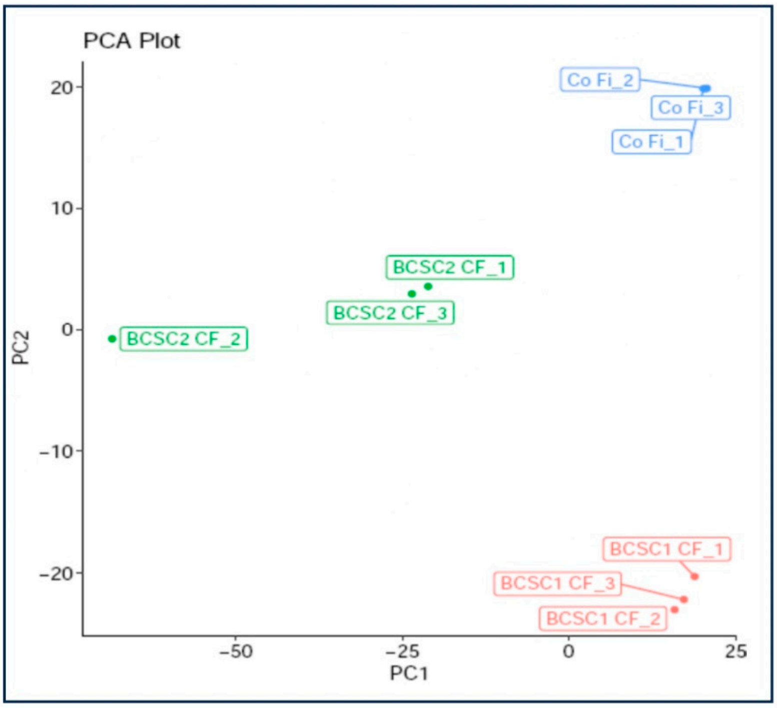

c

BCSC1 CF

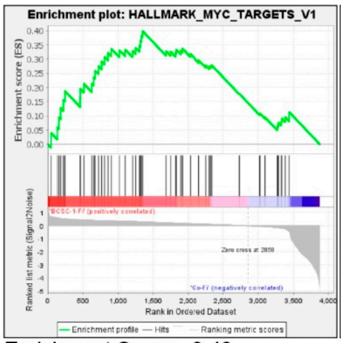

Enrichment Score: 0.40
P value: 0.000

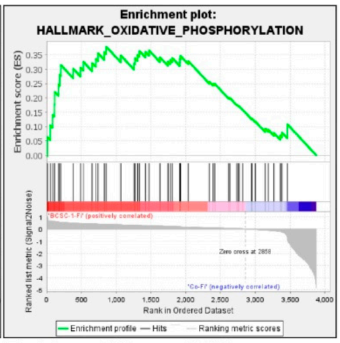

Enrichment Score: 0.37
P value: 0.03

BCSC2 CF

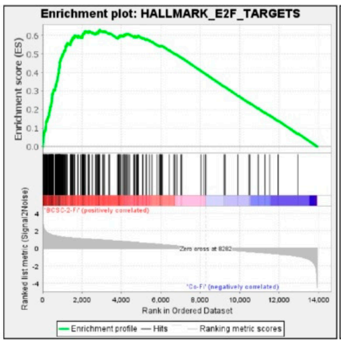

Enrichment Score: 0.63
P value: 0.000

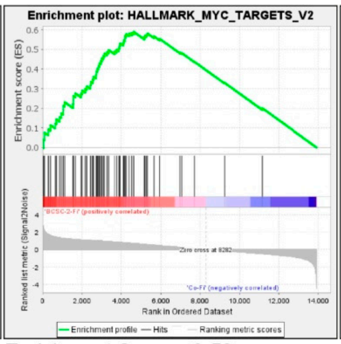

Enrichment Score: 0.58
P value: 0.000

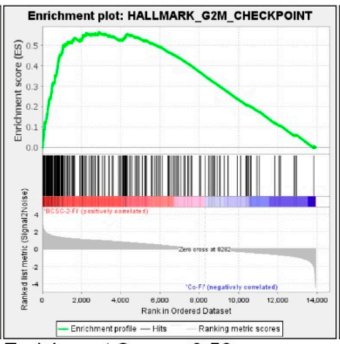

Enrichment Score: 0.56
P value: 0.000

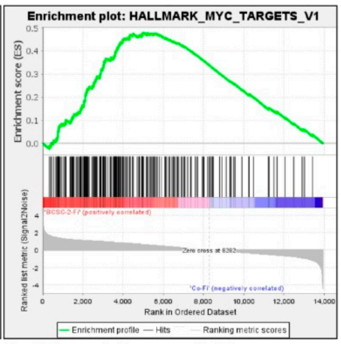

Enrichment Score: 0.47
P value: 0.000

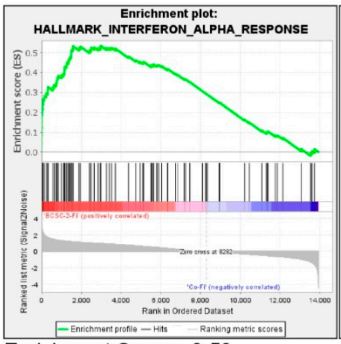

Enrichment Score: 0.53
P value: 0.000

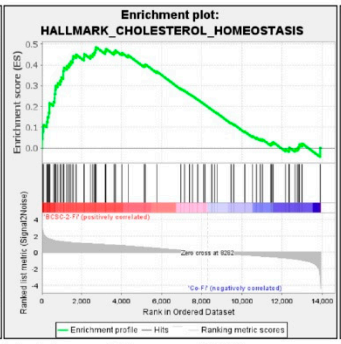

Enrichment Score: 0.49
P value: 0.000

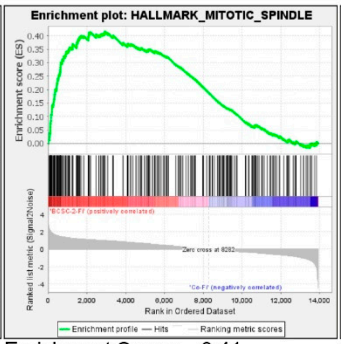

Enrichment Score: 0.41
P value: 0.000

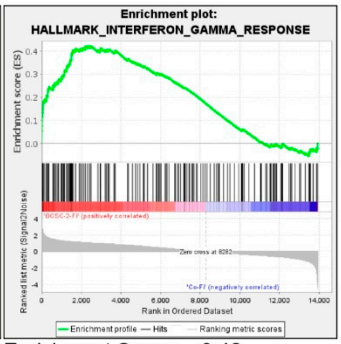

Enrichment Score: 0.42
P value: 0.000

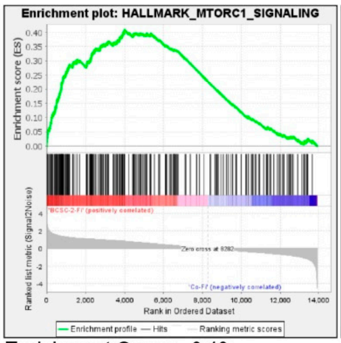

Enrichment Score: 0.40
P value: 0.000

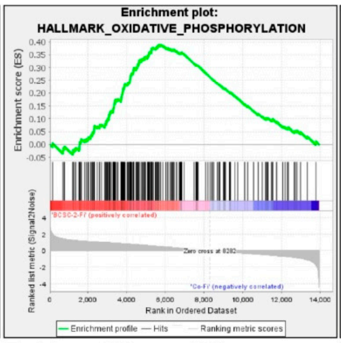

Enrichment Score: 0.38
P value: 0.000

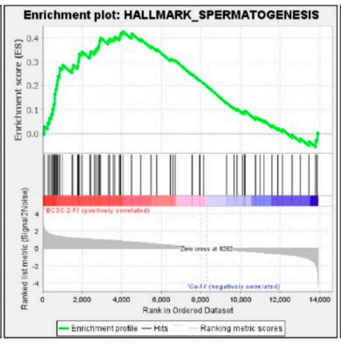

Enrichment Score: 0.42
P value: 0.001

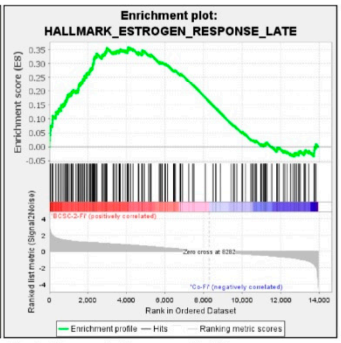

Enrichment Score: 0.36
P value: 0.000

BCSC2 CF

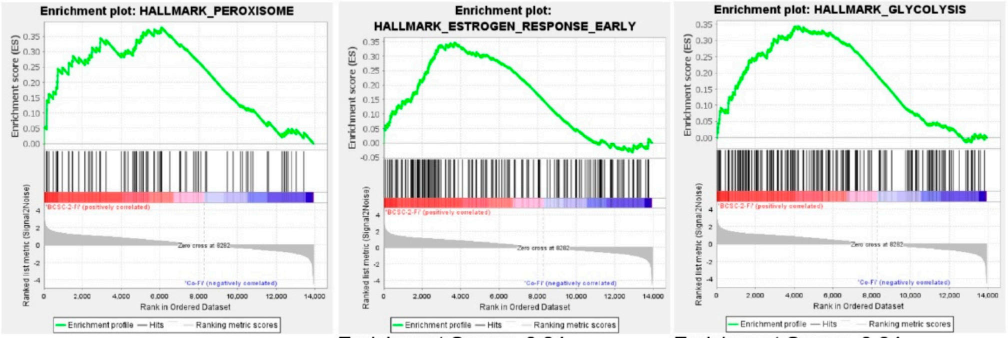

Enrichment Score: 0.37
P value: 0.02

Enrichment Score: 0.34
P value: 0.001

Enrichment Score: 0.34
P value: 0.004

D

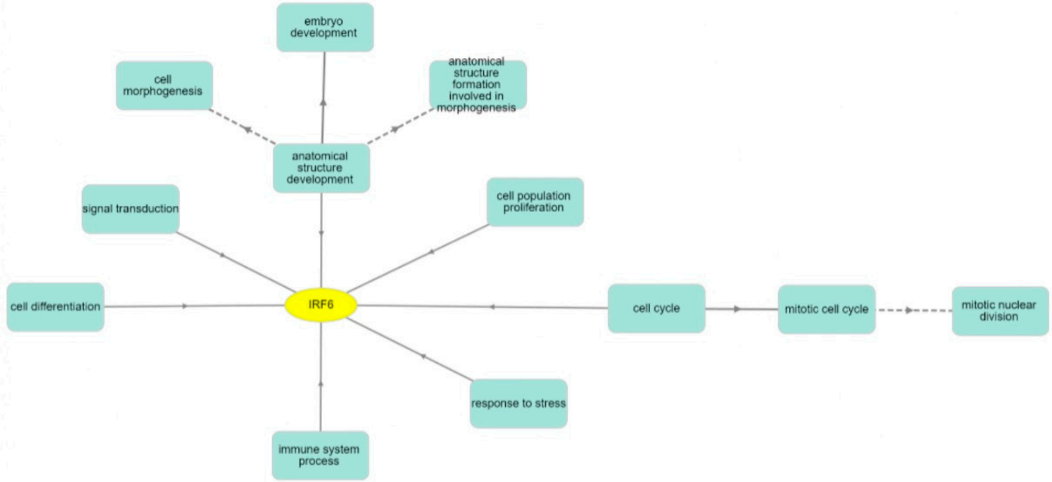

E

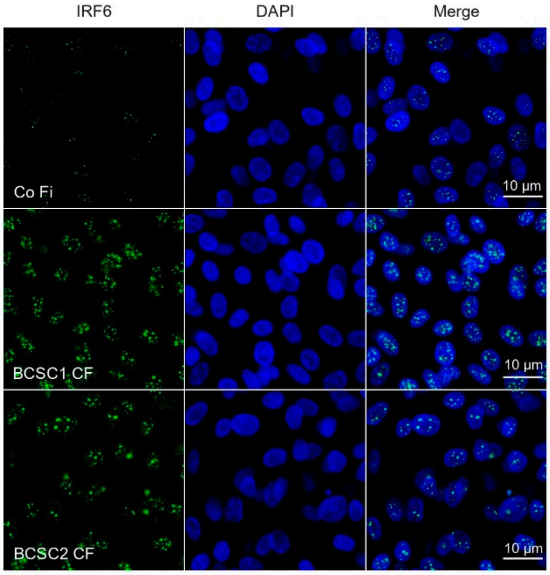

F

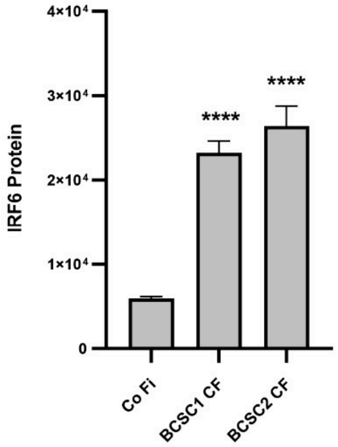

Figure S3:

(A) FACS plot of BCSC1 + Fi and BCSC2 + Fi co-cultures sorted for fibroblasts (RFP). **(B)** PCA plot indicating the variance in gene expression amongst the individual BCSC CF populations. **(C)** GSEA graphs of significantly enriched biological pathways in the BCSC1 CF and BCSC2 CF samples. **(D)** Commonly upregulated IRF6 gene in BCSC1 CF and BCSC2 CF populations is linked to

several biological processes. **(E)** Confocal images of BCSC + Fi co-cultures indicating nuclear localization of IRF6 gene (1:10 ratio). **(F)** Graphical representation of IRF6 protein quantification in BCSC1 CF and BCSC2 CF when compared to Co Fi using immunofluorescent images.

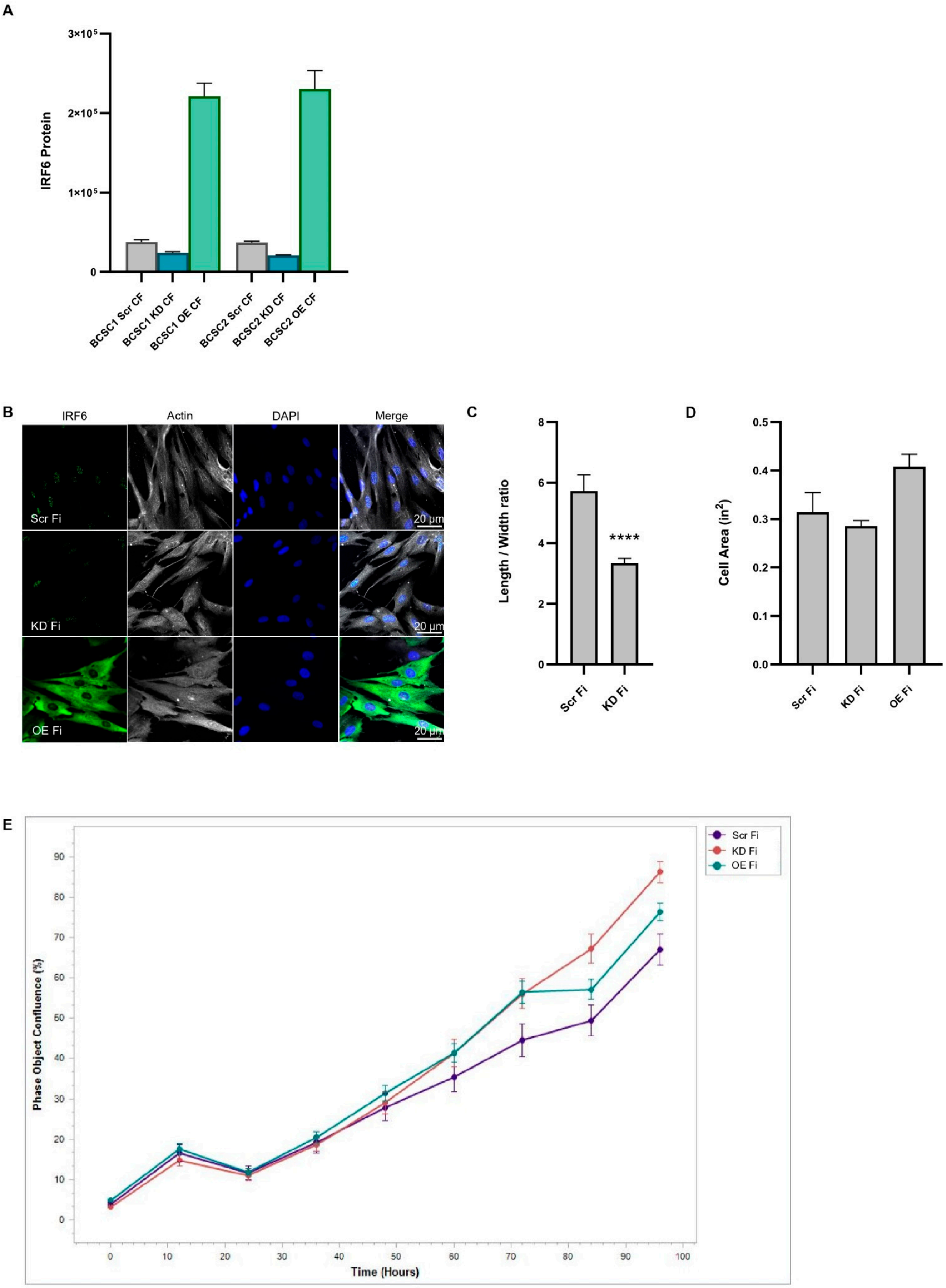

Figure S4:

(A) Graphical representation of IRF6 protein quantification in KD CF and OE CF when compared to Scr CF using immunofluorescent images (1:10 ratio). **(B)** Immunofluorescent images of Scr Fi, KD Fi and OE Fi depicting differences in cellular morphology, cell-cell adhesion and cell orientation **(C)** Graphical representation of the quantification of the length to width ratio of cells in Scr Fi and KD Fi cultures. **(D)** Graphical representation of cell surface area of Scr Fi, KD Fi and OE Fi. Statistical results are expressed as mean with SEM. Symbols applied: (*)-p ≤ 0.05; (**)-p ≤ 0.01; (***)-p ≤ 0.001; (****)-p ≤ 0.0001. **(E)** Proliferation curve of Scr Fi, KD Fi and OE Fi.

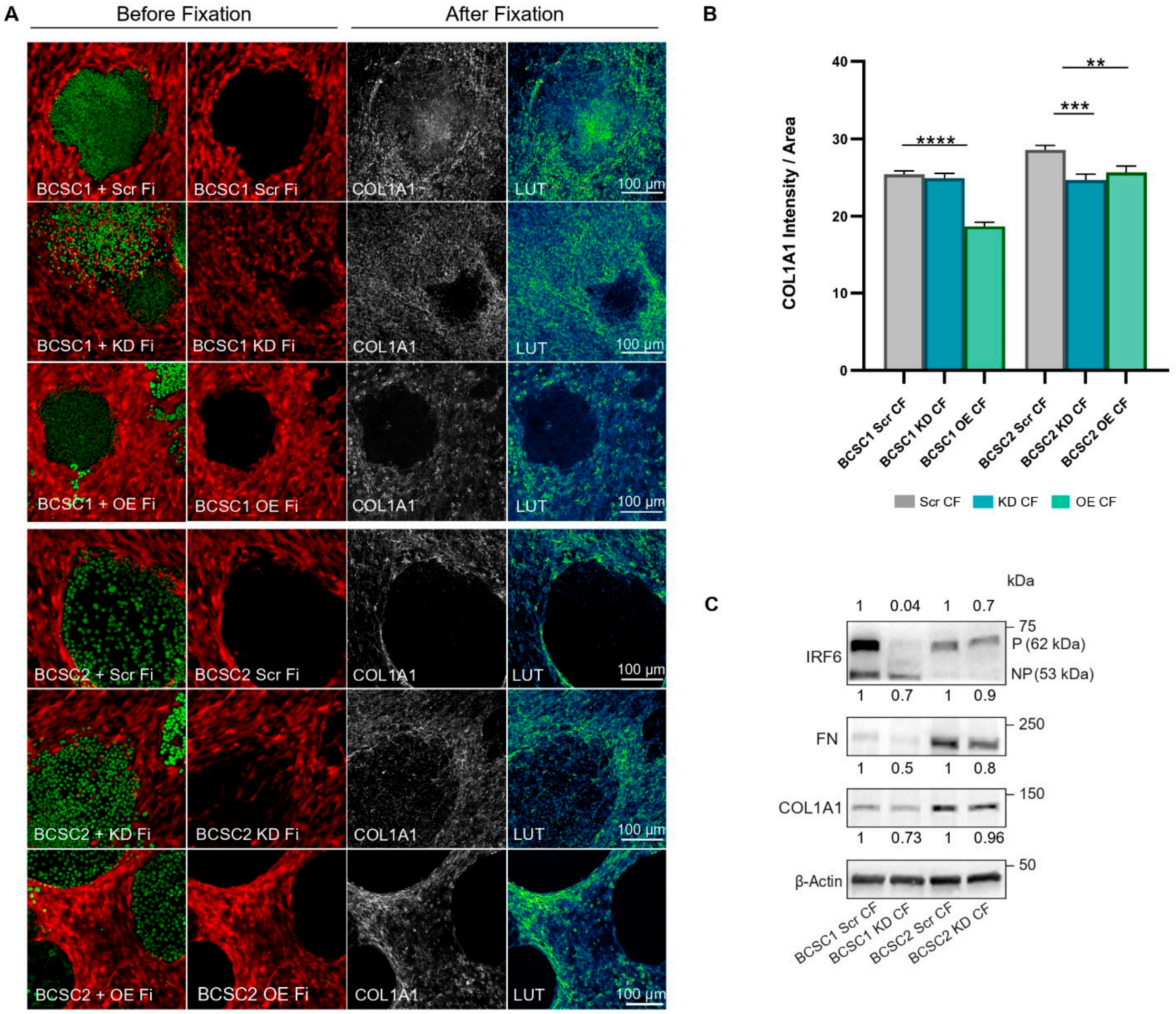

Figure S5:

(A) Confocal images representing collagen type 1 (COL1A1) deposition in BCSC + Scr Fi, BCSC + KD Fi and BCSC + OE Fi co-cultures (1:10 ratio). BCSC (EGFP); Fibroblast (RFP). Colour gradient (LUT) is from blue (low intensity signal) to yellow (high intensity signal). **(B)** Bar graph represents the measurement of collagen intensity by KD CF and OE CF in relation to Scr CF. **(C)** Western Blot data from BCSC Scr CF and BCSC KD CF indicating ECM expression (n=1).

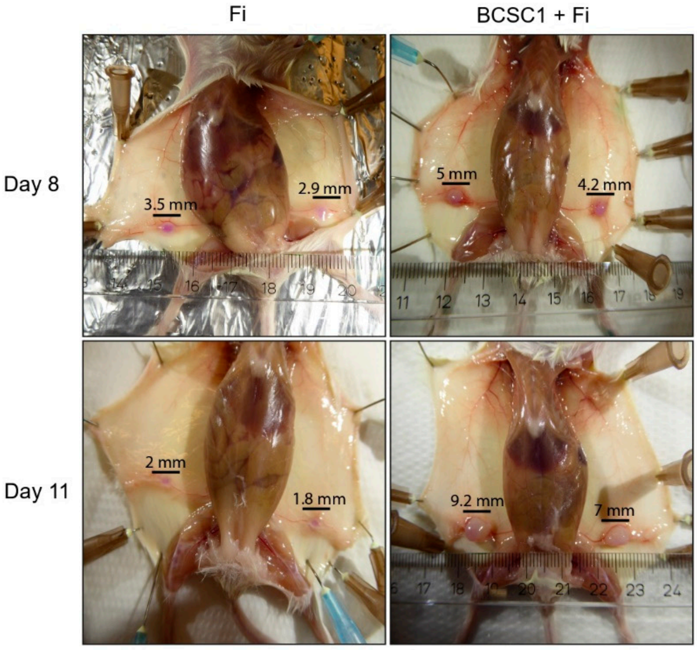

Figure S6:

Images showing the decrease in size of fibroblast (Fi) transplant observed on Day 8 and Day 11, when compared to the BCSC1/Fibroblast (BCSC1 + Fi) transplant (1:10) that has increased in size. Data indicates that the BCSC1 cells may induce survival signals in fibroblast cells to promote tumor growth.
